# Glucose transporter 4 promotes head and neck squamous cell carcinoma metastasis through the TRIM24-DDX58 axis

**DOI:** 10.1186/s13045-016-0372-0

**Published:** 2017-01-07

**Authors:** Yu-Chan Chang, Li-Hsing Chi, Wei-Ming Chang, Chia-Yi Su, Yuang-Feng Lin, Chi-Long Chen, Ming-Huang Chen, Peter Mu-Hsin Chang, Alex T. H. Wu, Michael Hsiao

**Affiliations:** 1 Graduate Institute of Life Sciences, National Defense Medical Center, Taipei, Taiwan; 2 Genomics Research Center, Academia Sinica, Taipei, Taiwan; 3 The Ph.D. Program for Translational Medicine, Taipei Medical University, Taipei, Taiwan; 4 Graduate Institute of Medical Sciences, National Defense Medical Center, Taipei, Taiwan; 5 Graduate Institute of Clinical Medicine, College of Medicine, Taipei Medical University, Taipei, Taiwan; 6 Department of Pathology, Taipei Medical University Hospital, Taipei Medical University, Taipei, Taiwan; 7 Department of Pathology, College of Medicine, Taipei Medical University, Taipei, Taiwan; 8 Department of Oncology, Taipei Veterans General Hospital, Taipei, Taiwan; 9 Faculty of Medicine, National Yang Ming University, Taipei, Taiwan; 10 Department of Biochemistry, College of Medicine, Kaohsiung Medical University, Kaohsiung, Taiwan

**Keywords:** GLUT4, HNSCC, TRIM24, DDX58, Metastasis

## Abstract

**Background:**

Head and neck squamous cell carcinoma (HNSCC) represents a unique and major health concern worldwide. Significant increases in glucose uptake and aerobic glycolysis have been observed in HNSCC cells. Glucose transporters (GLUTs) represent a major hub in the glycolysis pathway, with GLUT4 having the highest glucose affinity. However, GLUT4’s role in HNSCC has not been fully appreciated.

**Methods:**

An in silico analysis was performed in HNSCC cohorts to identify the most significant glucose transporter associated with HNSCC patient prognosis. An immunohistochemical analysis of a tissue microarray with samples from 90 HNSCC patients was used to determine the association of GLUT4 with prognosis. Complementary functional expression and knockdown studies of GLUT4 were performed to investigate whether GLUT4 plays a role in HNSCC cell migration and invasion in vitro and in vivo. The detailed molecular mechanism of the function of GLUT4 in inducing HNSCC cell metastasis was determined.

**Results:**

Our clinicopathologic analysis showed that increased GLUT4 expression in oral squamous cell carcinoma patients was significantly associated with a poor overall survival (OS, *P* = 0.035) and recurrence-free survival (RFS, *P* = 0.001). Furthermore, the ectopic overexpression of GLUT4 in cell lines with low endogenous GLUT4 expression resulted in a significant increase in migratory ability both in vitro and in vivo, whereas the reverse phenotype was observed in GLUT4-silenced cells. Utilizing a GLUT4 overexpression model, we performed gene expression microarray and Ingenuity Pathway Analysis (IPA) to determine that the transcription factor tripartite motif-containing 24 (TRIM24) was the main downstream regulator of GLUT4. In addition, DDX58 was confirmed to be the downstream target of TRIM24, whose downregulation is essential for the migratory phenotype induced by GLUT4–TRIM24 activation in HNSCC cells.

**Conclusions:**

Here, we identified altered glucose metabolism in the progression of HNSCC and showed that it could be partially attributed to the novel link between GLUT4 and TRIM24. This novel signaling axis may be used for the prognosis and therapeutic treatment of HNSCC in the future.

**Electronic supplementary material:**

The online version of this article (doi:10.1186/s13045-016-0372-0) contains supplementary material, which is available to authorized users.

## Background

Head and neck squamous cell carcinoma (HNSCC) ranks among the top ten cancers by occurrence worldwide [1]. For local HNSCC, recurrence and metastasis (R/M) have been regarded as the clinical factors associated with the poorest outcomes. Once a patient is diagnosed with R/M HNSCC, the prognosis is very poor, and the overall survival is often less than 1 year [2]. The underlying reasons for why relatively localized HNSCC becomes increasingly invasive and metastatic remain unclear and urgently need to be addressed. Previous reports have suggested that hypoxia could induce HNSCC cell migration and invasion [3, 4] and cause a switch to anaerobic glycolysis for energy and survival (known as the “Warburg effect”) [5]. This switch increases tumor cell proliferation rates by generating not only sufficient amounts of ATP but also high amounts of macromolecules [6]. In recent studies, such metabolic reprogramming has also been shown to contribute to cancer progression and metastasis [7]. However, how tumor cells establish this metabolic reprogramming and its influence on aggressive phenotypes are as yet unknown.

Glucose transporters (GLUTs) are membrane proteins that can facilitate glucose uptake and are found in most mammalian cells. There are 12 subtypes of GLUTs that have been identified in the human genome. Recently, the expression of GLUTs has been found in different cancers to modulate glucose metabolism and correlate with epithelial-mesenchymal transition (EMT) [8], chemotherapy resistance [9], and cell proliferation [10]. In this study, we first identified the expression of GLUT4 in oral squamous cell carcinoma and its prognostic impact on HNSCC patients. The overexpression of GLUT4 in the HNSCC cell lines Ca9-22 and HSC-3-M3 elevated the proliferation rate and migration ability. In vivo animal models validated that GLUT4-overexpressing HNSCC cells exhibited enhanced lymph node and lung metastasis. Finally, an in silico analysis found that the novel GLUT4–TRIM24 signaling pathway may contribute to these aggressive cancer phenotypes possibly through DDX58 downregulation.

## Methods

### Cell culture and stable clone establishment

The human head and neck squamous cancer cell lines FaDu, Detroit-562, HSC-2, HSC-3, HSC-M3, HSC-4, RPMI-650, and Ca-922 were grown in MEM supplemented with 10% FBS (Invitrogen, Carlsbad, CA, USA). All cells were incubated in a humidified atmosphere of 5% CO_2_ at 37 °C. All cell lines were purchased from the JCRB cell bank. The pGIPZ lentiviral shRNAmir system (Thermo, Waltham, MA, USA), virus-backboned short hairpin RNA (shRNA) clones, and the GLUT4 sequence were used to establish stable cell lines (Additional file 1: Table S5). Lentiviruses were used to infect the cells for 2 days. Stable clones were selected by treating the cells with 1 μg/ml puromycin (Sigma, St. Louis, MO, USA) for 2 weeks.

### Western blot analysis

HNSCC cell pellets were lysed in RIPA buffer with protease/phosphatase inhibitors on ice. The protein content was quantified using a BCA assay kit (Thermo, Waltham, MA, USA), and equal protein amounts (30 μg) of each sample were used for western blot analysis. PVDF membranes (Millipore, Bedford, MA, USA) were blocked with 5% fat-free milk and then incubated with primary antibodies directed against GLUT4 (Epitomics, Cambridge, MA, USA), GLUT1 (GeneTex, Hsinchu, Taiwan), DDX58 (GeneTex, Hsinchu, Taiwan) or OASL (GeneTex, Hsinchu, Taiwan), and α-tubulin (Sigma, St. Louis, MO, USA). Immunoreactive bands were visualized using an enhanced chemiluminescence (ECL) system (Amersham ECL Plus™, GE Healthcare Life Sciences, Chalfont St. Giles, UK).

### Microarray

Total RNA was extracted and purified using an RNeasy Mini kit (Qiagen, Valencia, CA, USA) and qualified with a model 2100 Bioanalyzer (Agilent Technologies, Palo Alto, CA, USA). All RNAs were labeled using a GeneChip 3′IVT Expression Kit & Hybridization Wash and Stain Kit (Affymetrix, Santa Clara, CA, USA) and analyzed using Affymetrix GeneChip Human Genome U133 plus 2.0 arrays (Affymetrix, Santa Clara, CA, USA). The gene expression levels were normalized as log2 values using GeneSpring software (Agilent Technologies, Palo Alto, CA, USA). Genes that were up- or downregulated with greater than 1.5-fold changes in response to GLUT4 overexpression were further subjected to computational simulation by Ingenuity Pathway Analysis (IPA; QIAGEN, Valencia, CA, USA) online tools to predict potential upstream regulators and canonical pathways. The microarray data were uploaded to the National Center for Biotechnology Information Gene Expression Omnibus (GEO, NCBI) (GSE89631).

### Glucose uptake and lactate production analyses and compounds

Glucose consumption and lactate production were measured using colorimetric glucose and lactate assay kits (BioVision, Milpitas, CA, USA) according to the manufacturer’s protocols. Briefly, cells from the designated experiments were incubated with assay buffer containing enzyme and glucose/lactate probes. Then, the optical densities were measured at 570/450 nm wavelengths. The glucose analog 2-(N-(7-nitrobenz-2-oxa-1,3-diazol-4-yl)amino)-2-deoxyglucose (2-NBDG; Sigma, St. Louis, MO, USA) was also used to analyze glucose uptake. In addition, cells were treated with the GLUT4 transport inhibitors indinavir or ritonavir (Sigma, St. Louis, MO, USA) at 100 and 50 μM, respectively, for 60 min, and the uptake of 2-NBDG was measured using Vector 3 (Bruker, MA, USA) to detect relative fluorescence counts.

### Immunohistochemical staining

Three representative 1-mm-diameter cores from each tumor, taken from formalin-fixed paraffin-embedded tissues, were selected for morphology typical of the diagnosis. Assessable cores were obtained in 90 cases. The histopathological diagnoses of all samples were reviewed and confirmed by a pathologist, Michael Hsiao. IHC staining was performed on serial 5-μm-thick tissue sections cut from the tissue microarray (TMA) using an automated immunostainer (Ventana, Tucson, AZ, USA). Briefly, the sections were first dewaxed in a 60 °C oven, deparaffinized in xylene, and rehydrated in graded alcohol. Antigens were retrieved by heat-induced antigen retrieval for 30 min in Tris-EDTA buffer. The slides were stained with a polyclonal rabbit anti-human GLUT4 antibody (1:750, Epitomics, Cambridge, MA, USA). The sections were subsequently counterstained with hematoxylin, dehydrated, and mounted. The IHC staining intensity was scored by two pathologists as follows: no cytoplasmic staining or cytoplasmic staining in <10% of tumor cells was defined as score 0; faint/barely perceptible partial cytoplasmic staining in >10% of tumor cells was defined as score 1+; moderate cytoplasmic staining in >10% of tumor cells was defined as score 2+; and strong cytoplasmic staining in >10% of tumor cells was defined as score 3+. Scores of 0 and 1+ were defined as low GLUT4 expression, while scores of 2+ and 3+ were defined as high GLUT4 expression.

### In vivo model

Age-matched, nonobese diabetic-severe combined immunodeficient gamma (NOD.Cg-Prkdc^scid^ Il2rg^tm1Wjl^/SzJ JAX®, NOD-SCID γ) male mice (6–8 weeks old, 20–25 g body weight) were used. To evaluate lung colony-forming ability, 1 × 10^6^ cells were resuspended in 100 μL of PBS and injected into the lateral tail vein. Lung nodule formation was quantified after H&E staining using a dissecting microscope at the endpoint. To evaluate in vivo tumorigenicity ability and establish an orthotopic model, 5 × 10^6^ cells were resuspended in 100 μL of PBS and then subcutaneously injected into the flanks of the mice, and 5 × 10^6^ cells were resuspended in 10 μL of PBS and injected into the buccal submucosa. All animal experiments were conducted in accordance with a protocol approved by the Academia Sinica Institutional Animal Care and Utilization Committee (IACUC).

### Case selection

In total, 90 patients diagnosed with head and neck squamous cell carcinoma at the Taipei Medical University Hospital in Taiwan from 1991 to 2010 were included in this study. Patients who received preoperative chemotherapy or radiation therapy were excluded. Clinical information and pathology data were collected via a retrospective review of patient medical records. All cases were staged according to the 7th edition of the Cancer Staging Manual of the American Joint Committee on Cancer (AJCC), and the histological cancer type was classified according to the World Health Organization (WHO) 2004 classification guidelines. Follow-up data were available in all cases, and the longest clinical follow-up time was 190 months. Overall survival and disease-free survival were defined as the intervals from surgery to death caused by head and neck squamous cell carcinoma and recurrence or distant metastasis, respectively. The study was performed with the approval of the Institutional Review Board and with permission from the ethics committee of the institution involved (TMU-IRB 99049).

### Statistical analysis

The nonparametric Mann–Whitney *U* test was used to analyze the statistical significance of results from three independent experiments. Statistical analyses were performed using SPSS (Statistical Package for the Social Sciences) 17.0 software (SPSS, Chicago, IL, USA). A paired *t* test was performed to compare the GLUT4 IHC expression levels in cancer tissues and in the corresponding normal adjacent tissues. The association between clinicopathological categorical variables and the GLUT4 IHC expression levels were analyzed by Pearson’s chi-square test. Estimates of the survival rates were calculated using the Kaplan–Meier method and compared using the log-rank test. The follow-up time was censored if the patient was lost during follow-up. Univariate and multivariate analyses were performed using Cox proportional hazards regression analysis with and without an adjustment for GLUT4 IHC expression level, tumor stage, lymph node stage, and recurrence status. For all analyses, a *P* value of <0.05 was considered significant.

## Results

### Increased expression of GLUT4 is significantly correlated with metastasis and poor prognosis in HNSCC patients

**Fig. 1:**
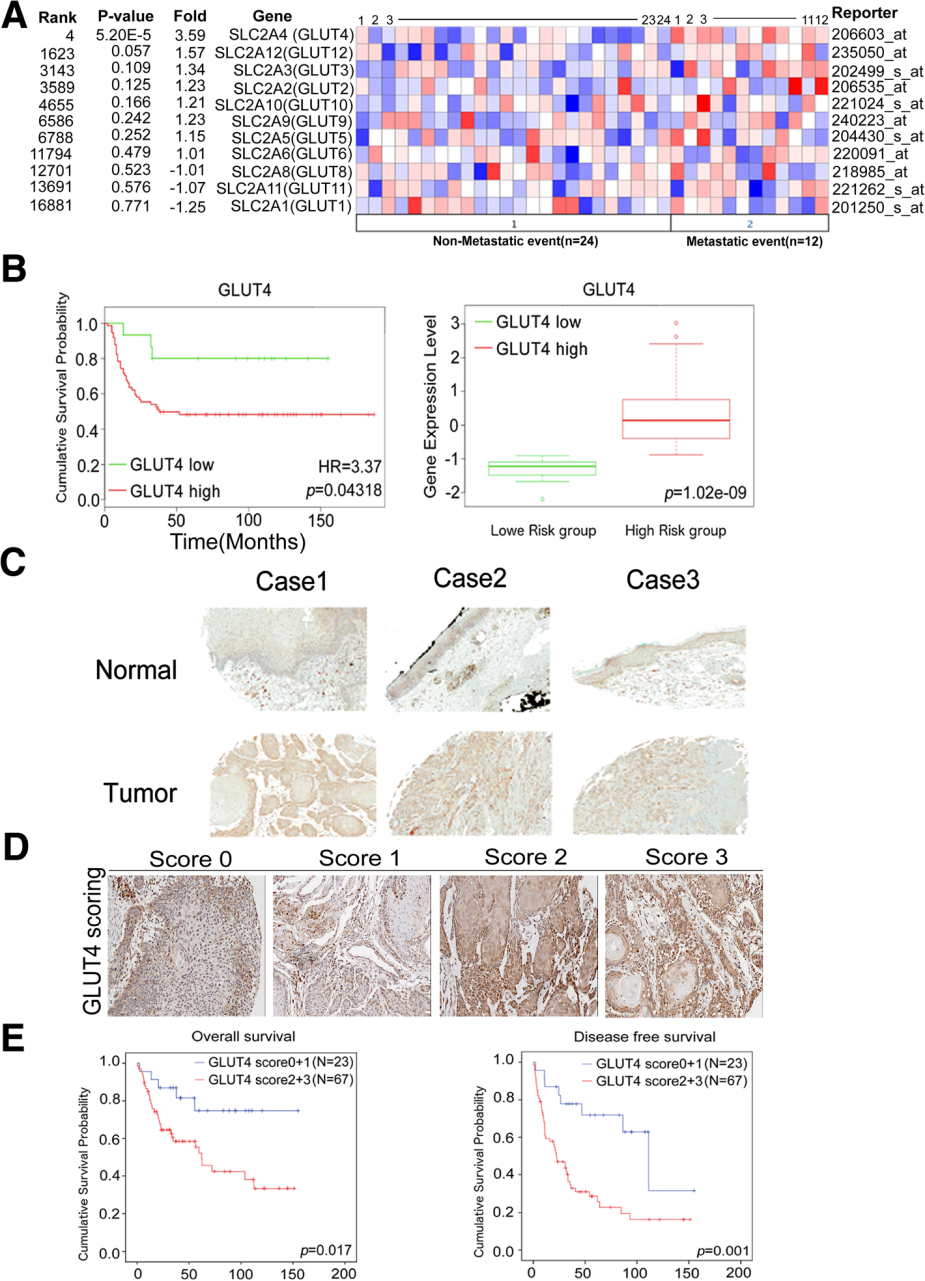
Overexpression of GLUT4 correlates with poor survival in HNSCC patients. **a** The heatmap indicates the correlation between the mRNA expression level of glucose transporters and HNSCC metastasis. Note that GLUT4 is the only gene that is significantly correlated with metastasis events in the Rickman Head–Neck cohort (*n* = 36) in the analysis by the Oncomine online tool. **b** The *box* plot shows that higher GLUT4 expression was correlated with a poor survival rate in patients in the Petel HNSCC cohort (E-MTAB-1328, *n* = 89) from the SurvExpress database (HR = 3.37, *P* = 0.043). **c** The expression level of the GLUT4 protein in tumor tissue compared to the corresponding normal adjacent tissue. **d** Scores (0~3) indicating GLUT4 levels in representative head and neck squamous tumor tissues. **e** Kaplan–Meier curves of overall and disease-free survival of 90 patients with HNSCC, stratified by a high or low GLUT4 protein expression level (*P* = 0.017 and *P* = 0.001, respectively)

To determine the clinical association between glucose transporters (GLUTs) in HNSCC patients, we utilized a previously developed HNSCC microarray database to examine and compare the expression of 10 major GLUTs using the Oncomine website. GLUT4 was found to be the only GLUT family member to have a significant correlation with metastatic status compared with other GLUT family members in the clinical cohort (Fig. 1a, 3.59-fold change, *P* = 5.20E-5). We then compared the correlations of all the GLUT family members with the prognosis of patients in the Petel HNSCC cohort (E-MTAB-1328, *n* = 89) in the SurvExpress database. The number of cases was divided approximately in half based on the expression (low or high) of the GLUT family member of interest, and a Kaplan–Meier survival analysis was performed on both groups using the SurvExpress website. The results showed that GLUT4 is the only GLUT family member whose RNA expression is significantly correlated with HNSCC overall survival (Fig. 1b, HR = 3.37, *P* value =0.043, other GLUT family data in Additional file 1: Figure S1). Forest plots of GLUT family members and their corresponding hazard ratios and Cox-*P* values were generated for another HNSCC microarray cohort (GSE2837, *n* = 40), and these results also showed GLUT4 to be the strongest prognosis marker with the highest hazard ratio. The Cox-*P* value for GLUT4 was calculated to be 0.07 by the Pronoscan website (Additional file 1: Figure S2). Together, these data show that of the GLUT family members, GLUT4 is the most significantly correlated with the clinical outcomes of HNSCC.

We next validated these findings by examining GLUT4 protein expression using our own clinical HNSCC tissue cohort. The immunohistochemical staining results showed stronger staining of the GLUT4 protein in tumor tissues than in the adjacent normal tissues (Fig. 1c). After scoring, we determined the correlation between patient survival and either low-level GLUT4 staining (Fig. 1d, IHC scores 0 and 1) or high-level GLUT4 staining (Fig. 1d, IHC scores 2 and 3). The results indicated that high-level GLUT4 staining was significantly correlated with the poor overall and disease-free survival probabilities (Fig. 1e, *P* = 0.017, *P* = 0.001, respectively). A clinicopathological analysis showed that high GLUT expression is significantly correlated with recurrence (Table 1, *P* = 0.001). The patient demographic features are shown in Additional file 1: Table S1. Univariate and multivariate analyses of the disease-free survival probability showed that high-level GLUT4 expression served as the strongest independent prognostic marker in both the univariate analysis (Table 2, HR = 3.35, *P* = 0.001) and the multivariate analysis (Table 2, HR = 3.76, *P* < 0.001). These data indicate that the upregulation of GLUT4 is significantly associated with the distant metastasis and disease-related progression in HNSCC patients.

**Table 1:** Correlation of clinicopathological features of HNSCC patients with GLUT4 expression

Clinicopathological feature	Number	GLUT4 expression, *n* (%)		*P*
		Low (*n* = 23)	High (*n* = 67)	
Age (years)				
<65	72	19 (26.4)	53 (73.6)	0.717
≥65	18	4 (22.2)	14 (77.8)	
Gender				
Male	81	22 (27.2)	59 (72.8)	0.295
Female	9	1 (11.1)	8 (88.9)	
T stage				
T1 + T2	64	17 (26.6)	47 (73.4)	0.731
T3 + T4	26	6 (23.1)	20 (76.9)	
N stage				
N0	63	16 (25.4)	47 (74.6)	0.958
N1–3	27	7 (25.9)	20 (74.1)	
M stage				
M0	88	23 (26.1)	65 (73.9)	0.402
M1	2	0 (00.0)	2 (100.0)	
Clinical stage				
I + II	48	13 (27.1)	35 (72.9)	0.825
III + IV	40	10 (25.0)	30 (75.0)	
Recurrence				
No	33	15 (45.5)	18 (54.5)	0.001*
Yes	57	8 (14.0)	49 (86.0)	

*P value <0.05 was considered statistically significant (Student’s *t* test for continuous variables and Pearson’s chi-square test for variables). SD represents the standard deviation. The tumor stage, tumor, lymph node, and distal metastasis status were classified according to the international system for staging HNSCC

**Table 2:** Univariate and multivariate analysis of GLUT4 expression and HNSCC patients

Variables		OS		DFS	
		HR (95% CI)	*P*	HR (95% CI)	*P*
Cox univariate analysis					
GLUT4 expression	High vs. low	2.99 (1.16–7.77)	0.02*	3.35 (1.62–7.30)	0.001*
T stage	T3–4 vs. T1–2	3.07 (1.61–5.84)	<0.001*	1.82 (1.05–3.17)	0.03*
N stage	N1–3 vs. N0	2.00 (1.04–3.85)	0.04*	1.69 (0.98–2.92)	0.06
Cox multivariate analysis					
GLUT4 expression	High vs. low	3.31 (1.28–8.55)	0.01*	3.76 (1.76–8.03)	<0.001*
T stage	T3–4 vs. T1–2	2.86 (1.47–5.54)	0.002*	1.80 (1.03–3.16)	0.04*
N stage	N1–3 vs. N0	1.72 (0.87–3.37)	0.1	1.73 (0.99–3.03)	0.06

*P value <0.05 was considered significant

### GLUT4 ectopic overexpression promotes the migration and invasion abilities of HNSCC cells

**Fig. 2:**
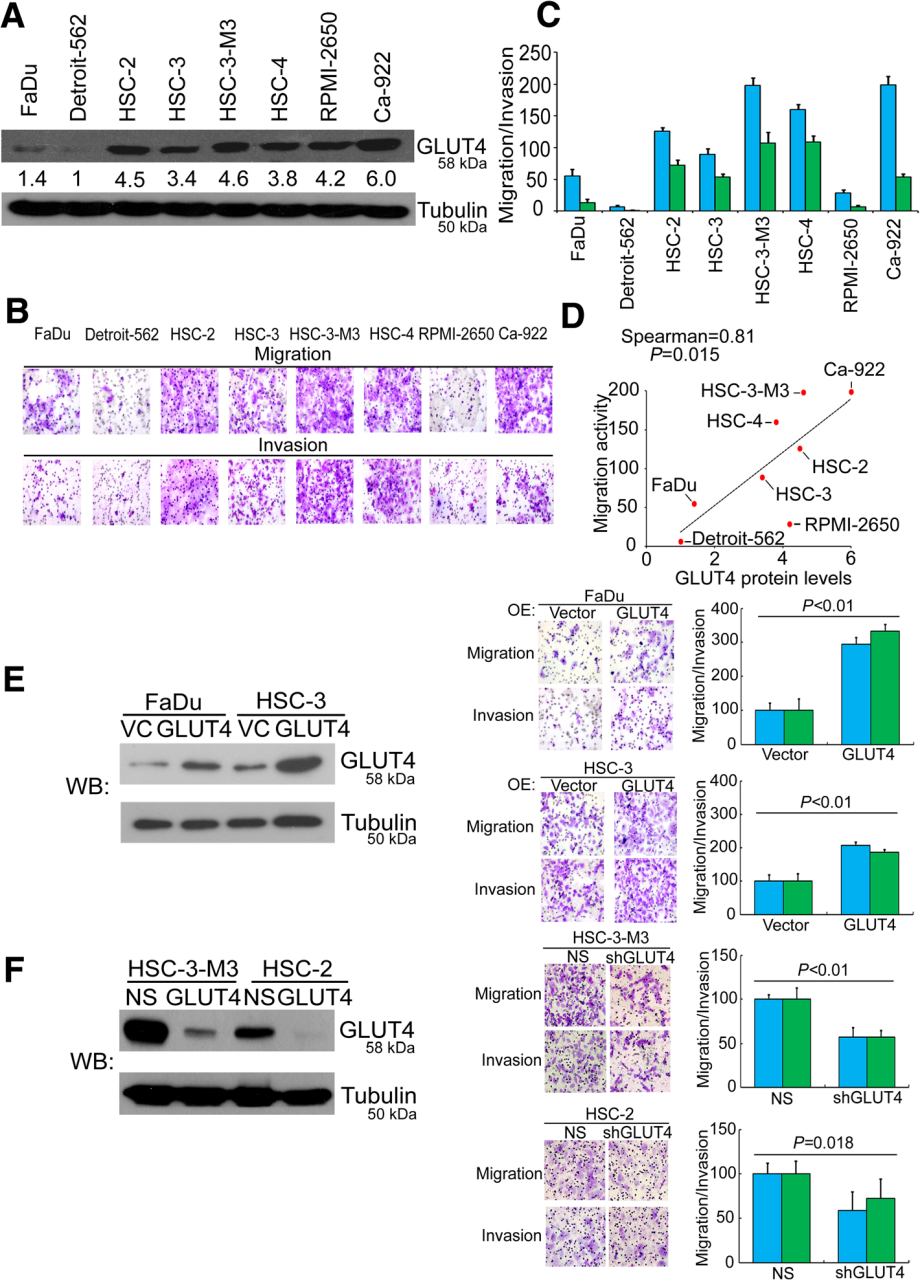
GLUT4 expression is positively correlated with metastasis ability in HNSCC cells and complementary models showed that GLUT overexpression promotes HNSCC migration and invasion. **a** Western blot analysis of GLUT4 and tubulin protein expression in various HNSCC cells. Tubulin was used as an internal control for protein loading. **b** The correlation between the GLUT4 protein expression level and the migration and invasion abilities of various HNSCC cell lines. **c** The significance of the correlation was analyzed using the nonparametric Spearman method. **d** Giemsa staining for evaluating the migration and invasion abilities of a panel of various HNSCC cell lines. **e** *Left panel*: western blot analysis of GLUT4 and tubulin protein expression after GLUT4 overexpression in FaDu cells and HSC-3 cells. Right panel: the migration and invasion abilities of FaDu cells and HSC-3 cells after the overexpression of the exogenous GLUT4 gene. **f** Western blot analysis of GLUT4 knockdown in HSC-2 cells and HSC-3-M3 cells. Tubulin was used as an internal control for protein loading. Right panel: the migration and invasion abilities of HSC-2 and HSC-3-M3 after GLUT4 knockdown. *NS* represents the nonsilenced control

To determine the functional attributes of GLUT4 in promoting HNSCC cellular migration and invasion, we first examined the GLUT4 protein expression levels in HNSCC cell lines. Our results showed varied expression levels of the GLUT4 protein in the eight HNSCC cell lines examined (Fig. 2a). We then determined the migration and invasion potentials of these HNSCC cell lines (Fig. 2b, c). The migration and invasion potentials of these cell lines were compared with their respective GLUT4 protein expression levels. Our results showed that GLUT4 expression appeared to be causally associated with metastatic potentials in HNSCC cells (Fig. 2d, Spearman rho = 0.81, *P* = 0.015). The GLUT4 gene was ectopically overexpressed in the low GLUT4-expressing cell lines HSC-3 and FaDu to determine whether GLUT4 overexpression induces HNSCC cell migration and invasion. The results in the left panel of Fig. 2e show the overexpression of the GLUT4 protein in the HSC-3 and FaDu cells. GLUT4 overexpression indeed significantly promoted the migration and invasion capabilities of the low-metastatic FaDu and HSC-3 cells (Fig. 2e, right panel, *P* < 0.01). In a complementary model, GLUT4 gene silencing significantly reduced the GLUT4 protein levels in HSC-3-M3 and HSC-2 cells, which expressed a high level of endogenous GLUT4 (Fig. 2f, left panel). GLUT4 knockdown in these two cell lines significantly inhibited the migratory/invasive capabilities of the highly metastatic HSC-3-M3 and HSC-2 cells (Fig. 2f, right panel).

### Increased GLUT4 expression promotes in vivo lung metastasis and in situ neck lymph node invasion

**Fig. 3:**
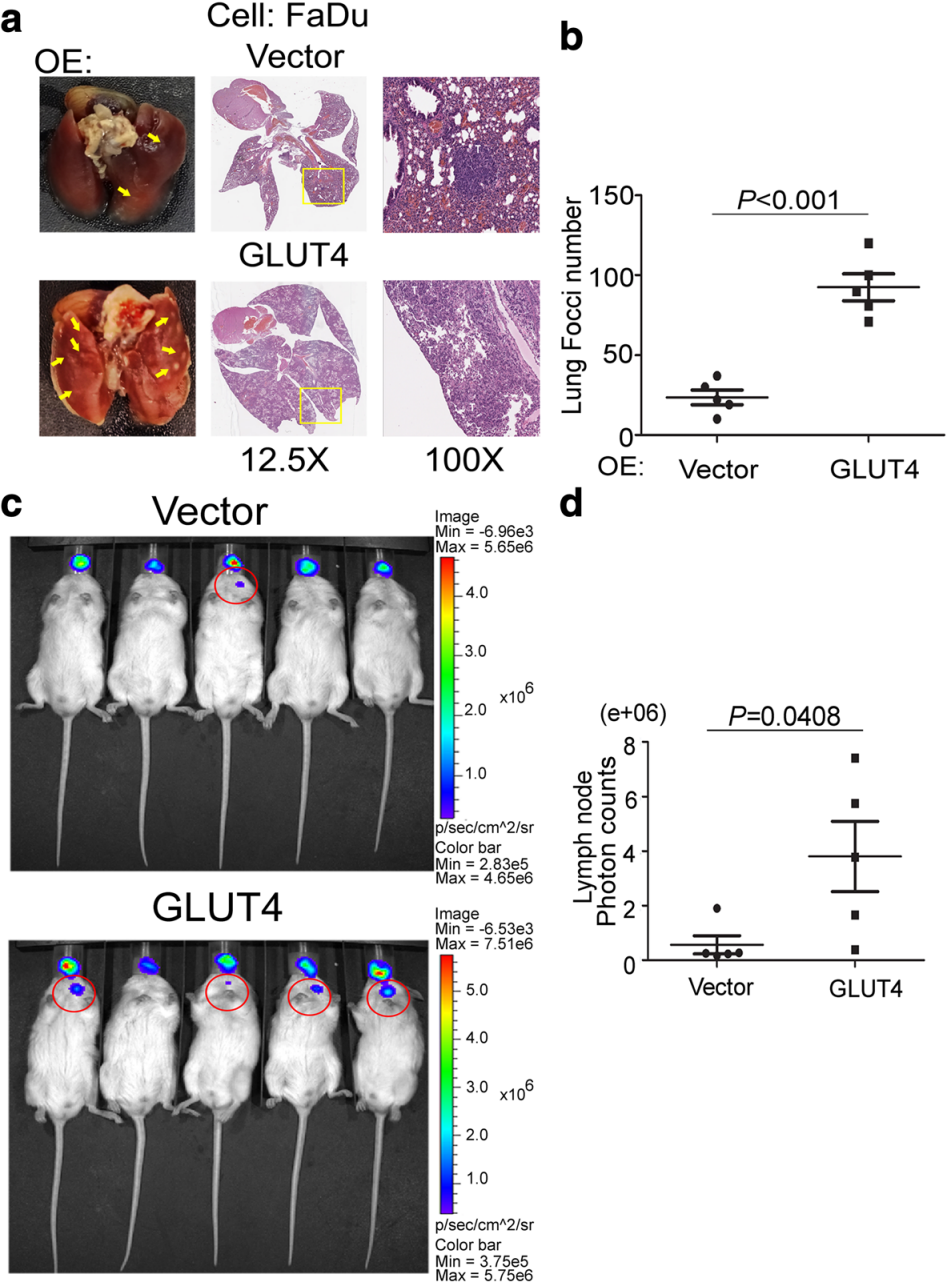
GLUT4 promotes in vivo metastasis and in situ invasion phenotypes. **a** Metastatic lung foci appearance as indicated by *arrows* (*left panel*) and foci morphologies (*middle panel*, ×12.5 magnification, and *right panel*, ×100 magnification) in mice (*n* = 5) implanted with control (vector only) or GLUT4-overexpressing FaDu cells through tail vein injection. **b** The quantified plot of metastatic lung foci numbers from Fig. 3a. **c** Bioluminescence images of the vector and GLUT4-overexpressed groups of the orthotopic FaDu xenograft mouse model. FaDu-GL-VC and –GLUT4 cells were intrabuccally injected into NSG mice. Luminescence was measured using a noninvasive bioluminescence imaging system (IVIS spectrum) at 6 weeks after injection. Lymph node metastasis is expressed as the bioluminescence intensity (BLI) change (five mice per group). **d** Quantitation of photon counts of each group from Fig. 3c. (*P* = 0.04). The significance of the difference was analyzed using the nonparametric Mann–Whitney *U* test

Next, we examined the role of GLUT4 in the promotion of metastasis in vivo using xenograft mouse models by intravenously injecting GLUT4-overexpressing and vector-control FaDu cells into mice. Six weeks after injection, the lungs were removed and examined for metastatic foci. Mice injected with GLUT4-overexpressing FaDu cells exhibited significantly higher numbers of metastatic foci compared to the vector control group by gross and histopathological examinations (Fig. 3a). There was a 4-fold increase in foci number in the GLUT4 overexpression group compared to the vector control group (Fig. 3b, *P* < 0.001). To mimic clinical HNSCC metastasis, we established an orthotopic xenograft HNSCC mouse model by injecting mice intrabuccally with luciferase-expressing FaDu cells that expressed either the GLUT gene or a vector control. Our results showed that stronger bioluminescence could be observed in 4 out of 5 mice injected with the GLUT4-overexpressing cells compared to only 1 mouse exhibiting weak bioluminescence in the vector control group (Fig. 3c). The average bioluminescence counts were obtained from the neck lymph nodes of all 10 mice, and the results showed that the GLUT4-overexpressing group had significantly higher counts compared to the vector control group (Fig. 3d, *P* < 0.05). In addition, we also established a xenograft model by subcutaneous injection of GLUT4-overexpressing FaDu cells. We observed that GLUT4 did not significantly increase the tumorigenicity of FaDu cells in vivo, and this result is consistent with the cell proliferation rate in vitro (Additional file 1: Figure S3). These data suggest that GLUT4 overexpression promotes HNSCC metastasis in vivo and in situ.

### HNSCC cell migration and invasion induced by GLUT4 overexpression is independent of glucose transporter activity

**Fig. 4:**
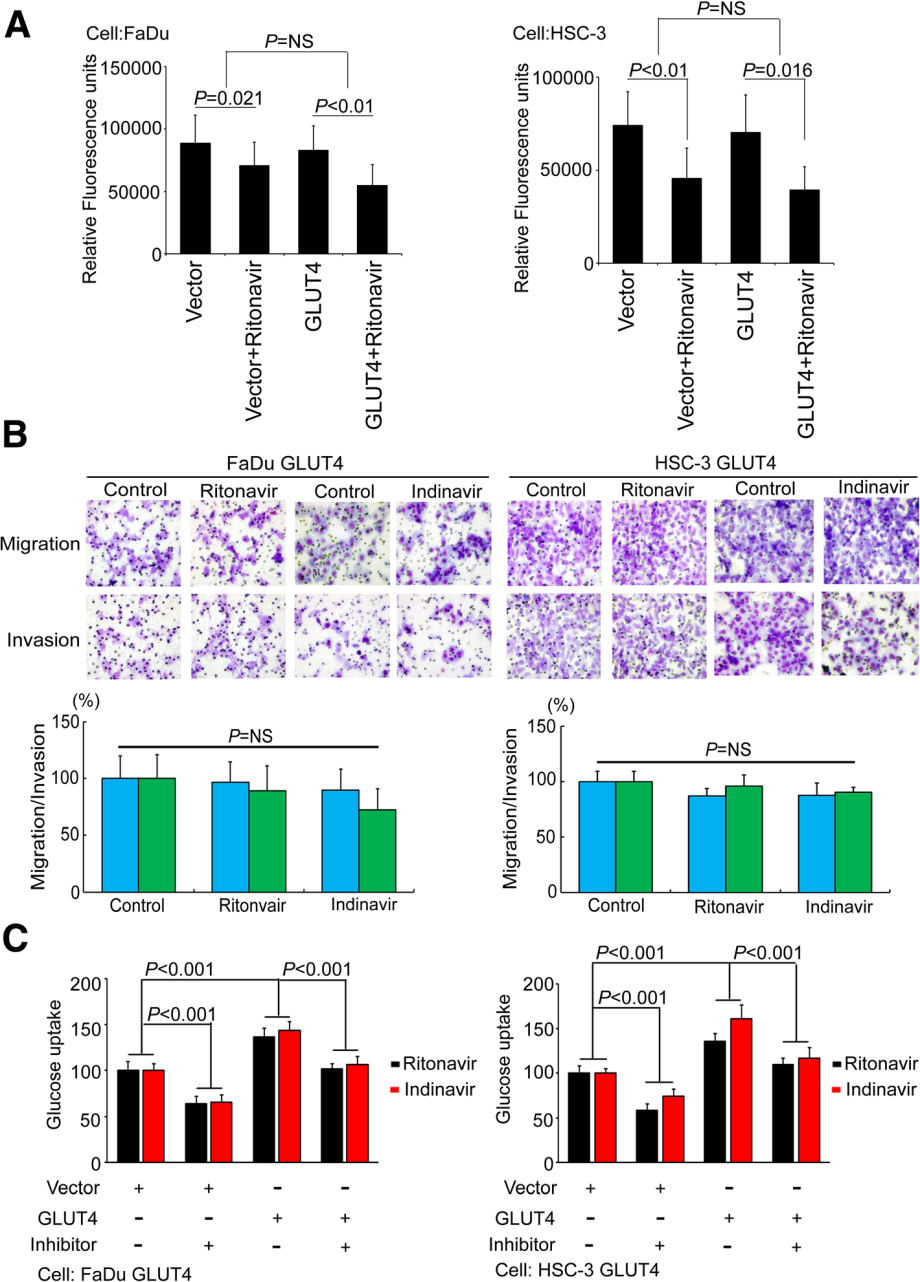
GLUT4 promotes HNSCC metastasis. **a** Relative fluorescence units after GLUT4 overexpression in FaDu (*left panel*) and HSC-3 cells (*right panel*) with or without ritonavir treatment. **b** The migration and invasion abilities of FaDu cells and HSC-3 cells were demonstrated after the overexpression of the exogenous GLUT4 gene, with and without the addition of ritonavir or indinavir. The data were the average of three independent experiments and are presented as the mean ± SEM. The significance of the difference was analyzed using the nonparametric Mann–Whitney *U* test. The *blue* and *green columns* represent cellular migration and invasion abilities, respectively. **c** The glucose uptake abilities of FaDu cells and HSC-3 cells were demonstrated after the overexpression of the exogenous GLUT4 gene, with and without the addition of ritonavir or indinavir. The data were the average of three independent experiments and are presented as the mean ± SEM. The significance of the difference was analyzed using the nonparametric Mann–Whitney *U* test. The *black* and *red columns* represent ritonavir and indinavir treatment, respectively

To determine whether the GLUT4-mediated promotion of HNSCC cell migration and invasion requires glucose transporter activity, we screened glucose uptake and lactate production in a panel of HNSCC cells. The data showed that metabolic events may be correlated with metastasis ability in several cell lines (Additional file 1: Figure S4), but no significant *P* values were obtained. Therefore, we added the glucose transport inhibitors ritonavir and indinavir to block glucose transport efficiency in a GLUT4-overexpressing cell model. We first used 2-NBDG treatment to demonstrate that ritonavir did indeed block transporter function. We observed the uptake of the glucose analog 2-NBDG by its autofluorescence. The GLUT4-overexpressing FaDu and HSC-3 cells treated with ritonavir had lower fluorescence counts (Fig. 4a, *P* < 0.01, left panel and *P* = 0.016, right panel) than did the vector-control FaDu and HSC-3 cells (Fig. 4a, *P* = 0.021, left panel and *P* < 0.01, right panel). We further confirmed the decrease in glucose uptake after inhibitor treatment by analyzing the culture medium (Fig. 4b). However, the results showed that ritonavir/indinavir did not significantly reduce the GLUT4-induced migration and invasion abilities of FaDu and HSC-3 cells compared to control cells (Fig. 4c). These results suggested that GLUT4 promotes HNSCC cell migration and invasion only partially through the transportation of glucose to the cancer cells.

### TRIM24-DDX58 axis is involved in GLUT4-mediated HNSCC cell migration

**Fig. 5:**
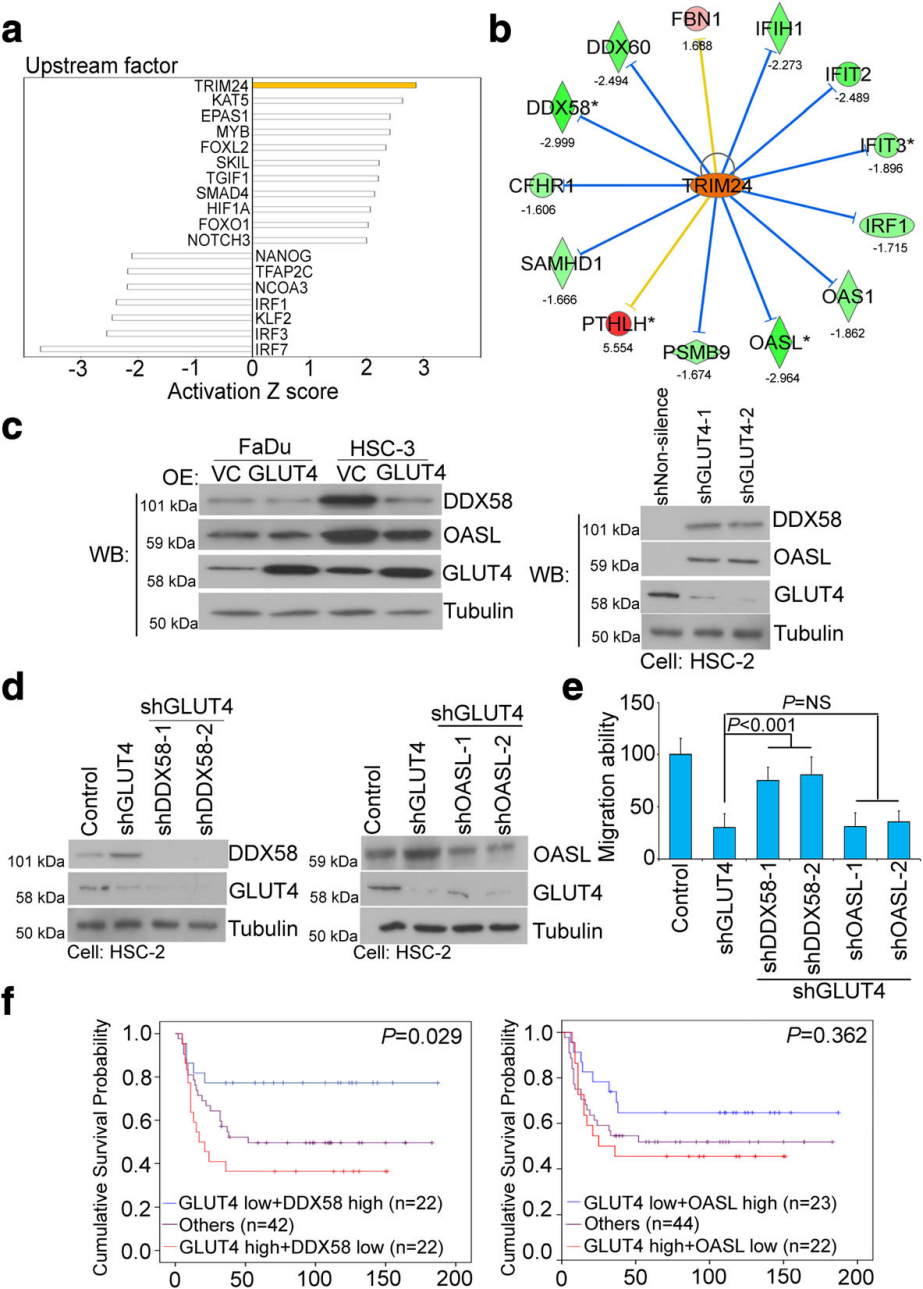
GLUT4 triggers TRIM24 activation to promote HNSCC metastasis. **a** The *bar chart* indicates the potential upstream regulators predicted by Ingenuity Pathway Analysis (IPA) software based on microarray from GLUT4-overexpressing FaDu cells with a 1.5-fold change cutoff compared to vector control cells. **b** The TRIM24 network was predicted based on the common signature from the Ingenuity (IPA) database overlaid with microarray data from GLUT4-overexpressing FaDu cells with a 1.5-fold change cutoff compared with vector control cells. The intensity of the node color indicates the degree of activating (*orange*) and inhibiting (*blue*) regulation following GLUT4 interactomics. **c** Western blot analysis of DDX58, OASL, and tubulin protein expression after GLUT4 overexpression in FaDu and HSC-3 cells (*left panel*) or GLUT4 knockdown in HSC-2 cells (*right panel*). Tubulin was used as an internal control for protein loading. **d** Western blot analysis of DDX58 or OASL knockdown combined with GLUT4 knockdown in HSC-2 cells. Tubulin was used as an internal control for protein loading. **e** The migration capabilities of HSC-2 cells with DDX58 or OASL knockdown combined with GLUT4 knockdown. **f** Kaplan–Meier survival curve analysis of HNSCC patients with high GLUT4 and low DDX58 or OASL levels as determined by IHC staining at the endpoint of overall survival (*P* = 0.029 and *P* = 0.362, respectively)

To determine whether another novel pathway or network by plays a transporter function-independent role in the GLUT4-mediated promotion of HNSCC cell migration and invasion, we next performed a microarray analysis using the low-metastatic HNSCC FaDu cells with or without GLUT4 overexpression. The normalized data from the microarray database analysis were subjected to Ingenuity Pathway Analysis (IPA) to identify molecules that are activated upon GLUT4 overexpression in FaDu cells. The results showed that the transcription factor TRIM24 is the top predicted candidate to be activated in response to GLUT4 overexpression, as verified by the transcriptional activity of its target genes with a *Z*-score of 2.868 and *P* value =1.55E-05 (Fig. 5a and Additional file 1: Table S2). The top 11 activated transcription factors with *Z*-scores higher than 2 and their respective downstream genes are shown in Additional file 1: Table S2. Similarly, the top 7 inhibited transcription factors and their respective downstream genes are shown in Additional file 1: Table S3.

Because TRIM24 was the most activated transcription factor upon GLUT4 overexpression in FaDu cells, we compared our GLUT4 microarray datasets with the TRIM24-related signature obtained by IPA analysis. Our results showed that DDX58 and OASL were the most significantly downregulated transcriptional targets of TRIM24 (Fig. 5b; −2.42- and −2.99-fold for DDX58 and −2.96- and −2.34-fold for OASL in Additional file 1: Table S4). We further validated the expression levels of DDX58 and OASL in cell models of GLUT4 overexpression and knockdown. Our western blot results showed that DDX58 and OASL were downregulated in GLUT4-overexpressing FaDu and HSC-3 cells (Fig. 5c, left panel) and that the knockdown of GLUT4 expression in HSC-2 cells resulted in the higher expression of the DDX58 and OASL proteins (Fig. 5c, right panel). We further knocked down the gene expression of DDX58 and OASL by their respective shRNAs in the GLUT4-knockdown highly metastatic HSC-2 cells. A western blot analysis showed that the DDX58 and OASL protein expression was significantly reduced (Fig. 5d). The subsequent knockdown of DDX58 could significantly restore the migration potential of GLUT4-knockdown HSC-2 cells by 1.5-fold (Fig. 5e, *P* < 0.001); however, OASL knockdown did not restore the migration capability (Additional file 1: Figure S5). These data suggested that DDX58 may be the primary negatively regulated downstream target of TRIM24 mediating the GLUT4-induced HNSCC cell migration. This in vitro inverse relationship of GLUT4 and DDX58 expression was then validated in in silico clinical HNSCC cohorts. The results showed that high GLUT4 RNA expression in combination with low DDX58 RNA expression levels was significantly correlated with the worst HNSCC patient survival (Fig. 5f and Additional file 1: Figure S6, *P* = 0.029, *P* < 0.001, respectively).

## Discussion

GLUT4, encoded by the SLC2A4 gene, is a high-capacity transporter that is normally restricted to nondividing cells, including adipose tissue, skeletal muscle, and myocardium [11]. GLUT4 is not detectable in normal oral epithelial cell lines [12], whereas GLUT1 is ubiquitously expressed and is constitutively located on the cell membrane [13]. Evidence from intensive research in the field of diabetes shows that GLUT4 traffics between the plasma membrane and intracellular vesicles (termed GLUT4-storage vesicles, GSVs) and that this activity is regulated by the PI3k/Akt pathway in an insulin-responsive manner [14] or by the AMPK pathway [15] in response to muscle contraction. Surprisingly, evidence has suggested that GLUT4 is present for basal glucose consumption and cell growth and survival in multiple myeloma [10] and breast cancer cells [16]. To date, little is known about the involvement of GLUT4 in cancer metabolism. This raises the question of whether the regulation of GLUT4 in cancer cells is due to a cancer-specific glucose transporter or a cancer-specific signaling mechanism. In this study, we first confirmed the role of GLUT4 in cancer metastasis and the possible signaling network involved.

TRIM24 controls gene expression through several mechanisms. First, TRIM24 promotes AKT phosphorylation to promote cell proliferation [17]. Second, TRIM24 interacts with nuclear receptor, such as RAR or ER, to regulate gene expression [18]. Third, TRIM24 contains a RING domain and E3 ligase activity that degrades p53, which controls gene expression [19]. Because our signaling analysis was generated using GLUT4-silenced HNSCC cells, we proposed that GLUT4 triggers TRIM24 to repress several downstream tumor suppressors. We hypothesized that the GLUT4–TRIM24 axis had a positive correlation and represented a powerful biomarker for clinical treatment and prognosis.

One of the most interesting observations we made in this study was that GLUT4-mediated HNSCC metastasis was independent of glucose concentration and the innate glucose transport function of GLUT4. It may be that the ectopic overexpression of GLUT4 leads to a lower threshold for activating its downstream molecules, rendering the ligand (glucose) concentration nonconsequential. This phenomenon was reported in the case of epidermal growth factor (EGF) and its receptor (EGFR) [20–22]. It is also plausible that alternative ligands (other than glucose) of GLUT4 may be present and responsible for this phenomenon. Thus, further investigation is required.

Based on its tissue and function specificity, GLUT4 has long been thought to be an insulin-dependent glucose transporter in muscle and fat cells. Our study thus uncovers a new role for GLUT4 as a metastatic promoter and prognostic biomarker for HNSCC patients. However, the reason why GLUT4 expression is elevated in HNSCC cells remains unclear. A plausible explanation could be the deranged metabolism in cancer cells. Because the upper aerodigestive tract is susceptible to environmental carcinogens, such as tobacco, alcohol, and betel nuts [23], cellular stress and damages generated by these agents may result in malignant transformation and metabolism. Our IPA analysis revealed that hypoxia-induced factors (EPAS1, HIF1A) and TGF*B*-associated genes (SKIL, TGIF1, SMAD4) as well as genes involved in stemness and tumorigenesis (MYB, FOXL2, FOXO1, NOTCH3) were all upregulated, which supported the hypothesis that GLUT4 may be an abnormal responder to environmental carcinogens and result in carcinogenesis, cancer progression, and metabolic shifts. (Fig. 5a and Additional file 1: Table S2).

According to recently published reports, TRIM24 was found to be correlated with poor survival and was involved in cell proliferation and metastasis in colon cancer and breast cancer [24, 25]. TRIM24 was also reported to be a regulator of interferon signal transducers to activate the STAT pathway through retinoic acid receptor inhibition [26, 27]. TRIM24 serves as a co-factor for binding the *STAT1* promoter region to enhance cell proliferation through the induction of the IFN/STAT1 pathway [26, 28]. Interestingly, DDX58 was found to be significantly upregulated in TRIM24-deficient mice [27]. Here, in our study, we further provided direct evidence that GLUT4 overexpression significantly activates TRIM24 to downregulate DDX58 expression and consequently promotes HNSCC cell motility and invasion. The detailed mechanism regarding how GLUT4 modulates TRIM24 activity remains to be elucidated.

## Conclusions

In this study, we showed that GLUT4 overexpression promotes tumor metastasis and is significantly associated with poor prognosis in HNSCC patients through a glucose-indirect pathway in cancer cells that leads to the activation of the TRIM24 pathway. Furthermore, we validated the downstream target DDX58 as the suppressor of GLUT4–TRIM24-induced migration and invasion. The inverse correlation of GLUT4 and DDX58 may be used as a significant predictor of poor prognosis in HNSCC patients. The GLUT4–TRIM24 axis may serve as a new target for drug development to treat HNSCC patients with metastasis.

## Additional files

Additional file 1: Table S1. Demographic features of HNCC patient cohort. **Table S2.** GLUT overexpression activated transcription factors and their downstream targets ranked by *Z*-Score. **Table S3.** GLUT overexpression inhibited transcription factors and their downstream targets ranked by *Z*-Score. **Table S4.** List of TRIM24 downstream genes and their fold changes. **Table S5.** List of primers and knockdown clones’ information. **Table S6.** List of candidate probes >2.0-fold change cutoff by GLUT4 vs. control in FaDu cells. **Figure S1.** Box plot showing the expression of the GLUT family members correlated with the survival rate of the patients in the Petel HNSCC cohort (E-MTAB-1328, *n* = 89) in the SurvExpress database (HR = 3.37, *P* = 0.043). **Figure S2.** Forest plot of GLUT family members and their corresponding hazard ratios, probes and Cox-P values. **Figure S3.** GLUT4 overexpression model in vitro and in vivo. (A) Cell proliferation rate and (B) tumorigenicity ability in animal model GLUT4-overexpressing FaDu cells. **Figure S4.** Glucose uptake and lactate production in a panel of HNSCC cell lines. **Figure S5.** The migration abilities of with or without GLUT4 knockdown combined DDX58 or OASL knockdown in HSC-2 cells. **Figure S6.** Correlation plot of GLUT4 expression with the (A) OASL or (B) DDX58 RNA level in a clinical cohort (Pearson *r* = −0.7146, *P* < 0.001 and Pearson *r* = −0.6246, *P* < 0.001, respectively). (DOCX 2697 kb)

## Abbreviations

2-NBDG: (2-(N-(7-nitrobenz-2-oxa-1,3-diazol-4-yl)amino)-2-deoxyglucose)
DDX58: DEXD-H-box helicase 58
GLUT: Glucose transporter
IPA: Ingenuity Pathway Analysis
OASL: 2′-5′-Oligoadenylate synthetase Like
TRIM24: Tripartite motif-containing 24
