# Supplementary material for: Glucose transporter 4 promotes head and neck squamous cell carcinoma metastasis through the TRIM24-DDX58 axis
Source: J Hematol Oncol. 2017 Jan 7;10:11. doi: 10.1186/s13045-016-0372-0 (PMC5219690; doi:10.1186/s13045-016-0372-0)

**Glucose Transporter 4 Promotes Head and Neck Squamous Cell Carcinoma Metastasis through the TRIM24-DDX58 Axis**

Yu-Chan Chang^1,2^, Li-Hsing Chi^2,3^, Wei-Ming Chang^2.4^, Chia-Yi Su^2^, Yuang-Feng Lin^5^, Chi-Long Chen^6,7^, Ming Huang Chen^8,9^, Peter Mu-Hsin Chang^8,9#^, Alex T.H. Wu^3,#^ and Michael Hsiao^2,10#^

1. Graduate Institute of Life Sciences, National Defense Medical Center, Taiwan

2. Genomics Research Center, Academia Sinica, Taipei, Taiwan

3. The Ph.D. Program for Translational Medicine, Taipei Medical University, Taipei, Taiwan.

4. Graduate Institute of Medical Sciences, National Defense Medical Center, Taiwan

5. Graduate Institute of Clinical Medicine, College of Medicine, Taipei Medical University, Taipei, Taiwan.

6. Department of Pathology, Taipei Medical University Hospital, Taipei Medical University, Taipei, Taiwan

7. Department of Pathology, College of Medicine, Taipei Medical University, Taipei, Taiwan

8. Division of Hematology and Oncology, Department of Medicine, Taipei Veterans General Hospital, Taipei, Taiwan

9. Faculty of Medicine, National Yang Ming University, Taipei, Taiwan

10. Department of Biochemistry, College of Medicine, Kaohsiung Medical University, Kaohsiung, Taiwan

# Correspondence to Dr. Michael Hsiao

Genomics Research Center, Academia Sinica, Taipei, Taiwan.

E-mail: [mhsiao@gate.sinica.edu.tw](mailto:mhsiao@gate.sinica.edu.tw)

Or to Dr. Alex TH Wu

The Ph.D. Program for Translational Medicine, Taipei Medical University, Taipei, Taiwan.

E-mail: [chaw1211@tmu.edu.tw](mailto:chaw1211@tmu.edu.tw)

Or to Dr. Peter Mu-Hsin Chang

Division of Hematology and Oncology, Department of Medicine, Taipei Veterans General Hospital, Taipei, Taiwan

E-mail: [i54821413@yahoo.com.tw](mailto:i54821413@yahoo.com.tw)

**Running Title**: GLUT4 overexpression promotes head and neck squamous cell carcinoma metastasis

**Supplementary Materials and Methods**

**RT-PCR Analysis**

Cells were lysed in TRIzol reagent (Invitrogen, Carlsbad, CA, USA), and total RNA was extracted according to the manufacturer’s protocol. The amount of RNA was measured using a Nanodrop spectrophotometer (Thermo, Waltham, MA, USA). Reverse transcription-PCR (RT-PCR) was performed using a SuperScript III kit (Invitrogen, Carlsbad, CA, USA) according to the manufacturer’s protocol. The expression levels of target genes were normalized to that of the ribosomal protein 26S, which was used as an internal control.

**Cell Migration and Invasion Assays**

For migration assays, polycarbonate filters (GE Healthcare Life Sciences, Chalfont St. Giles, UK) were coated with 1 mg/ml human fibronectin (Sigma, St. Louis, MO, USA). Medium containing 10% FBS was added to each well of the lower compartment of the Boyden chamber. Cells were resuspended in serum-free medium and loaded into each uncoated well of the upper compartment of the Boyden chamber. For invasion assays, the fibronectin-coated polycarbonate filters were further coated with 10 mg/ml Matrigel (BD Biosciences, San Jose, CA, USA) on the opposite surface. Medium containing 10% FBS was added to each well of the lower compartment of the Boyden chamber. Cells were resuspended in serum-free medium and loaded into each Matrigel-coated well of the upper compartment of the Boyden chamber. After an optimized length of time, the invaded cells were stained with Giemsa solution and counted under a light microscope (400x, 8 random fields of each well). Three independent experiments were conducted in quadruplicate assays.

**Supplementary Tables**

**Table S1.** Demographic features of HNCC patient cohort

| Clinicopathological feature | n |
| --- | --- |
|  |  |
| Total number | 90 |
| Age |  |
| <65 y | 72 |
| ≧65 y | 18 |
| Gender |  |
| Male | 81 |
| Female | 9 |
| T stage |  |
| T1+T2 | 64 |
| T3+T4 | 26 |
| N stage |  |
| N0 | 63 |
| N1-3 | 27 |
| M stage |  |
| M0 | 88 |
| M1 | 2 |
| Clinical stage |  |
| I + II | 48 |
| III + IV | 40 |
| Recurrence |  |
| No | 33 |
| Yes | 57 |

**Table S2.** GLUT overexpression activated transcription factors and their downstream targets ranked by Z-Score.

**Table S3.** GLUT overexpression inhibited transcription factors and their downstream targets ranked by Z-Score.

**Table S4.** List of TRIM24 downstream genes and their fold changes

| Gene Symbol | Fold Change | Regulation | Probe Set ID |
| --- | --- | --- | --- |
| DDX58 | -2.4163973 | down | 218943_s_at |
| DDX58 | -2.9992216 | down | 222793_at |
| OASL | -2.9635885 | down | 205660_at |
| OASL | -2.3428178 | down | 210797_s_at |
| DDX60 | -2.4938993 | down | 218986_s_at |
| IFIT2 | -1.4442453 | down | 217502_at |
| IFIT2 | -2.4892886 | down | 226757_at |
| IFIH1 | -1.0672822 | down | 216020_at |
| IFIH1 | -2.272543 | down | 219209_at |
| IFIT3 | -1.7283401 | down | 204747_at |
| IFIT3 | -1.8957736 | down | 229450_at |
| SAMHD1 | -1.6664855 | down | 234987_at |
| SAMHD1 | -1.4990468 | down | 235529_x_at |
| SAMHD1 | -1.3548536 | down | 235964_x_at |
| SAMHD1 | -1.3361026 | down | 1559883_s_at |
| OAS1 | -1.86244 | down | 202869_at |
| OAS1 | -1.1846541 | down | 205552_s_at |
| IRF1 | -1.0984706 | down | 202531_at |
| IRF1 | -1.715033 | down | 238725_at |
| PSMB9 | -1.674197 | down | 204279_at |
| CFHR1 | -1.6062323 | down | 215388_s_at |
| PTHLH | 4.2370477 | up | 206300_s_at |
| PTHLH | 3.111123 | up | 210355_at |
| PTHLH | 3.1474395 | up | 211756_at |
| FBN1 | 1.1511298 | up | 202765_s_at |
| FBN1 | 1.6881784 | up | 202766_s_at |
| FBN1 | 1.1516863 | up | 235318_at |

**Table S5.** List of primers and knockdown clones’ information.

| **Primer** | **Sequence** |  |  |
| --- | --- | --- | --- |
| GLUT4-F | CTGTGCCATCCTGATGACTG |  |  |
| GLUT4-R | CGTAGCTCSTGGCTGGAACT |  |  |
| S26RT-F | CCGTGCCTCCAAGATGACAAAG |  |  |
| S26RT-R | ACTCAGCTCCTTACATGGGCTT |  |  |
| CDH1-F | AGTGCCAACTGGACCATTCA |  |  |
| CDH1-R | TCTTTGACCACCGCTCTCCT |  |  |
| Snail-F | GCGAGCTGCAGGACTCTAAT |  |  |
| Snail-R | TCCAAGGAAGAGGCTGAAGTA |  |  |
| Slug-F | GAGCATACAGCCCCATCACT |  |  |
| Slug-R | GCAGTGAGGGCAAGAAAAAG |  |  |
| Vimentin-F | ACGTGACTACGTCCACCCGCA |  |  |
| Vimentin-R | CTCCTCCTGCAATTTCTCCCGGAGG |  |  |
| TWIST1-F | CGGACAAGCTGAGCAAGATT |  |  |
| TWIST1-R | CCTTCTCTGGAAACAATGA |  |  |
| **shRNA** | **Oligo ID** | **NM ID** | **Target sequence** |
| shGLUT4-1 | TRCN0000043631 | 001042 | CCGGCTCCTTCCTCATTGGTATCATCTCGAGATGATACCAATGAGGAAGGAGTTTTTG |
| shGLUT4-2 | TRCN0000043632 | 001042 | CCGGGTGATTGAACAGAGCTACAATCTCGAGATTGTAGCTCTGTTCAATCACTTTTTG |
| shDDX58-1 | V2LHS_197765 | 014314 |  |
| shDDX58-2 | V2LHS_197176 | 014314 |  |
| shOASL-1 | V2LHS_3221 | 198213 |  |
| shOASL-2 | V2LHS_24137 | 198213 |  |

**Table S6.** List of candidate probes >2.0 fold change cut-off by GLUT4 versus control in FaDu cells.

| Probe Set ID | FC (FaDu GLUT4 vs FaDu VC) | Log FC (FaDu GLUT4 vs FaDu VC) | Regulation (FaDu GLUT4 vs FaDu VC) | Gene Symbol |
| --- | --- | --- | --- | --- |
| 1555756_a_at | -5.6320996 | -2.4936728 | down | CLEC7A |
| 226237_at | -4.2286477 | -2.0801964 | down | COL8A1 |
| 235456_at | -3.9463327 | -1.9805126 | down |  |
| 215071_s_at | -3.9144719 | -1.9688177 | down | HIST1H2AC |
| 236193_at | -3.69586 | -1.88591 | down | HIST1H2BC |
| 213418_at | -3.4645748 | -1.7926784 | down | HSPA6 |
| 228425_at | -3.316558 | -1.7296867 | down | PAX8-AS1 |
| 221698_s_at | -3.2812095 | -1.7142277 | down | CLEC7A |
| 228328_at | -3.2644892 | -1.7068572 | down | KLHL28 |
| 225275_at | -3.2462163 | -1.6987591 | down | EDIL3 |
| 209911_x_at | -3.1995518 | -1.6778698 | down | HIST1H2BD |
| 223690_at | -3.090091 | -1.6276493 | down | LTBP2 |
| 214455_at | -3.0765982 | -1.621336 | down | HIST1H2BC///HIST1H2BE///HIST1H2BF///HIST1H2BG///HIST1H2BI |
| 239430_at | -3.0259123 | -1.5973701 | down | IGFL1 |
| 222793_at | -3.0004885 | -1.5851974 | down | DDX58 |
| 236175_at | -2.9721353 | -1.5714998 | down | TRIM55 |
| 205660_at | -2.9635258 | -1.5673146 | down | OASL |
| 242931_at | -2.9566948 | -1.5639853 | down |  |
| 206696_at | -2.9537616 | -1.5625534 | down | GPR143 |
| 219660_s_at | -2.9438944 | -1.5577259 | down | ATP8A2 |
| 208982_at | -2.9224017 | -1.5471544 | down | PECAM1 |
| 201466_s_at | -2.8553321 | -1.5136585 | down | JUN |
| 237515_at | -2.8043184 | -1.4876502 | down | TMEM56 |
| 209925_at | -2.795469 | -1.4830904 | down | OCLN |
| 238439_at | -2.780564 | -1.4753776 | down | ANKRD22 |
| 220169_at | -2.775192 | -1.4725876 | down | TMEM156 |
| 231849_at | -2.7560947 | -1.4626255 | down | KRT80 |
| 205190_at | -2.6689525 | -1.4162736 | down | PLS1 |
| 230493_at | -2.6175826 | -1.3882351 | down | SHISA2 |
| 204967_at | -2.5737953 | -1.3638973 | down | SHROOM2 |
| 204682_at | -2.5477574 | -1.3492279 | down | LTBP2 |
| 210387_at | -2.535288 | -1.3421497 | down | HIST1H2BG///HIST1H2BJ |
| 219863_at | -2.533035 | -1.340867 | down | HERC5 |
| 202291_s_at | -2.5323687 | -1.3404875 | down | MGP |
| 236133_x_at | -2.5084324 | -1.326786 | down | ZNF254 |
| 219659_at | -2.4954774 | -1.3193159 | down | ATP8A2 |
| 218986_s_at | -2.4930785 | -1.3179283 | down | DDX60 |
| 226757_at | -2.488053 | -1.3150172 | down | IFIT2 |
| 244163_at | -2.4785428 | -1.3094921 | down | SEMA3A |
| 220979_s_at | -2.4766872 | -1.3084116 | down | ST6GALNAC5 |
| 230356_at | -2.4681165 | -1.3034105 | down | RP13-238F13.5 |
| 230795_at | -2.4566638 | -1.2967005 | down |  |
| 227112_at | -2.4555786 | -1.296063 | down | TMCC1 |
| 208981_at | -2.4463322 | -1.2906203 | down | PECAM1 |
| 236128_at | -2.4411669 | -1.287571 | down | ZNF91 |
| 231248_at | -2.4196749 | -1.2748132 | down | AX747517///CST6 |
| 202803_s_at | -2.419674 | -1.2748127 | down | ITGB2 |
| 218943_s_at | -2.4165156 | -1.2729282 | down | DDX58 |
| 226535_at | -2.4051623 | -1.2661343 | down | ITGB6///LOC100505984 |
| 238825_at | -2.3988445 | -1.2623396 | down | ACRC |
| 227492_at | -2.3784378 | -1.2500143 | down | OCLN |
| 208083_s_at | -2.3682137 | -1.2437992 | down | ITGB6///LOC100505984 |
| 234980_at | -2.3634515 | -1.2408953 | down | TMEM56 |
| 213352_at | -2.3591263 | -1.2382526 | down | TMCC1 |
| 201578_at | -2.3442624 | -1.2291341 | down | PODXL |
| 210797_s_at | -2.340827 | -1.2270184 | down | OASL |
| 219424_at | -2.3404448 | -1.2267828 | down | EBI3 |
| 232151_at | -2.334317 | -1.2230005 | down | MACC1 |
| 1568812_at | -2.3282323 | -1.219235 | down | LOC100507140 |
| 1555878_at | -2.3220665 | -1.2154093 | down | RPS24 |
| 224917_at | -2.318758 | -1.2133522 | down | MIR21///VMP1 |
| 1405_i_at | -2.3118477 | -1.2090464 | down | CCL5 |
| 203438_at | -2.3064609 | -1.2056808 | down | STC2 |
| 205969_at | -2.292986 | -1.1972275 | down | AADAC |
| 229787_s_at | -2.2818298 | -1.1901913 | down | OGT |
| 239500_at | -2.279002 | -1.1884022 | down | EFCAB1 |
| 219209_at | -2.2719533 | -1.1839333 | down | IFIH1 |
| 205992_s_at | -2.2301183 | -1.1571202 | down | IL15 |
| 227489_at | -2.2172291 | -1.1487579 | down | SMURF2 |
| 224341_x_at | -2.1915674 | -1.131963 | down | TLR4 |
| 229372_at | -2.1908789 | -1.1315098 | down | GOLT1A |
| 209469_at | -2.183807 | -1.1268454 | down | GPM6A |
| 1554406_a_at | -2.171683 | -1.1188135 | down | CLEC7A |
| 230689_at | -2.1693313 | -1.1172504 | down |  |
| 202149_at | -2.1655116 | -1.114708 | down | NEDD9 |
| 203535_at | -2.1607416 | -1.1115265 | down | S100A9 |
| 226374_at | -2.142915 | -1.0995746 | down | CXADR |
| 1563075_s_at | -2.1369858 | -1.0955772 | down |  |
| 230660_at | -2.1362627 | -1.095089 | down | SERTAD4 |
| 225032_at | -2.1203592 | -1.0843086 | down | FNDC3B///LOC101928615 |
| 1555759_a_at | -2.1167207 | -1.081831 | down | CCL5 |
| 201860_s_at | -2.110783 | -1.0777783 | down | PLAT |
| 1566766_a_at | -2.1099076 | -1.0771799 | down | MACC1 |
| 211668_s_at | -2.1045706 | -1.0735259 | down | PLAU |
| 213281_at | -2.1004372 | -1.0706897 | down | JUN |
| 214587_at | -2.0952334 | -1.067111 | down | COL8A1 |
| 207046_at | -2.0936413 | -1.0660143 | down | HIST1H4A///HIST1H4B///HIST1H4C///HIST1H4D///HIST1H4E///HIST1H4F///HIST1H4H///HIST1H4I///HIST1H4J///HIST1H4K///HIST1H4L///HIST2H4A///HIST2H4B///HIST4H4 |
| 202489_s_at | -2.0903652 | -1.063755 | down | FXYD3 |
| 201525_at | -2.088571 | -1.0625162 | down | APOD |
| 202936_s_at | -2.0859702 | -1.0607185 | down | SOX9 |
| 228345_at | -2.0802088 | -1.0567284 | down | CHIC1 |
| 201428_at | -2.0652497 | -1.0463161 | down | CLDN4 |
| 206118_at | -2.062873 | -1.0446548 | down | STAT4 |
| 235146_at | -2.0624828 | -1.0443821 | down | TMCC3 |
| 229796_at | -2.0605478 | -1.0430279 | down | SIX4 |
| 1553829_at | -2.0574646 | -1.0408676 | down | CYP1B1-AS1 |
| 205404_at | -2.0447564 | -1.031929 | down | HSD11B1 |
| 227345_at | -2.043416 | -1.030983 | down | TNFRSF10D |
| 231964_at | -2.041525 | -1.0296471 | down | BICD1 |
| 227314_at | -2.0403547 | -1.02882 | down | ITGA2 |
| 204174_at | -2.0342245 | -1.0244789 | down | ALOX5AP |
| 226155_at | -2.0280938 | -1.0201244 | down | FAM160B1 |
| 224558_s_at | -2.024662 | -1.0176811 | down | MALAT1 |
| 219764_at | -2.0180318 | -1.012949 | down | FZD10 |
| 227949_at | -2.0155156 | -1.0111489 | down | PHACTR3 |
| 239638_at | -2.01315 | -1.0094547 | down |  |
| 220104_at | -2.0107243 | -1.0077152 | down | ZC3HAV1 |
| 205479_s_at | -2.001664 | -1.0011997 | down | PLAU |
| 225163_at | -2.001646 | -1.0011868 | down | FRMD4A |
| Probe Set ID | **FC (FaDu GLUT4 vs FaDu VC)** | **Log FC (FaDu GLUT4 vs FaDu VC)** | **Regulation (FaDu GLUT4 vs FaDu VC)** | **Gene Symbol** |
| 236451_at | 2.0001216 | 1.0000877 | up | LOC100996579 |
| 232371_at | 2.0013385 | 1.0009651 | up | 7-Mar |
| 1560910_at | 2.004159 | 1.0029969 | up | PPIL6 |
| 212992_at | 2.0132859 | 1.009552 | up | AHNAK2 |
| 1559725_at | 2.0147595 | 1.0106077 | up | LOC101928555 |
| 232184_at | 2.0252678 | 1.0181127 | up | ALS2 |
| 242467_at | 2.027327 | 1.0195789 | up |  |
| 1556814_a_at | 2.0273275 | 1.0195792 | up | CTA-292E10.6 |
| 1554324_s_at | 2.0284357 | 1.0203676 | up | DYNC2LI1 |
| 209351_at | 2.0301676 | 1.0215988 | up | KRT14 |
| 1554520_at | 2.030838 | 1.0220752 | up | LOC283861 |
| 1560208_at | 2.0308769 | 1.0221028 | up | LOC101930593///LOC102724352 |
| 1555007_s_at | 2.0349865 | 1.0250192 | up | WDR66 |
| 240382_at | 2.0361416 | 1.0258379 | up | LOC101928076 |
| 220550_at | 2.0378544 | 1.027051 | up | FBXO4 |
| 229338_at | 2.039388 | 1.0281363 | up | LOC100289361 |
| 226166_x_at | 2.0400865 | 1.0286303 | up | STK36 |
| 231863_at | 2.0421197 | 1.0300674 | up | ING3 |
| 1559534_at | 2.0429907 | 1.0306826 | up | BC040901///CTC-241N9.1///LOC100996419 |
| 238946_at | 2.0434134 | 1.0309811 | up |  |
| 227406_at | 2.043997 | 1.031393 | up | GABPB1-AS1 |
| 218789_s_at | 2.0442395 | 1.0315642 | up | C11orf71 |
| 239739_at | 2.0469391 | 1.0334682 | up | SNX24 |
| 1557432_at | 2.0469391 | 1.0334682 | up | RASAL2 |
| 1556147_at | 2.0469396 | 1.0334685 | up | RP11-16N11.2 |
| 231085_s_at | 2.0519545 | 1.0369987 | up |  |
| 210078_s_at | 2.0521498 | 1.0371361 | up | KCNAB1 |
| 212374_at | 2.0538857 | 1.0383558 | up | FEM1B |
| 231954_at | 2.0540981 | 1.0385051 | up | DKFZP434I0714///MIR4453 |
| 236780_at | 2.0561528 | 1.0399475 | up |  |
| 214157_at | 2.0588527 | 1.0418406 | up | GNAS |
| 237502_at | 2.0622973 | 1.0442524 | up | CRLS1 |
| 1557179_s_at | 2.0657108 | 1.0466383 | up | CARS2 |
| 201744_s_at | 2.0770643 | 1.0545459 | up | LUM |
| 210462_at | 2.0797317 | 1.0563974 | up | BLZF1 |
| 238529_at | 2.0805314 | 1.056952 | up | RP11-196G18.23 |
| 232615_at | 2.0812094 | 1.0574222 | up |  |
| 230998_at | 2.0824947 | 1.0583129 | up | CBX3 |
| 1561924_at | 2.0824962 | 1.0583138 | up |  |
| 236752_at | 2.0856035 | 1.0604649 | up |  |
| 238029_s_at | 2.0891469 | 1.0629139 | up | SLC16A14 |
| 210095_s_at | 2.0895584 | 1.0631981 | up | IGFBP3 |
| 1567032_s_at | 2.0896497 | 1.063261 | up | ZNF160 |
| 236982_at | 2.0912387 | 1.0643578 | up |  |
| 1567224_at | 2.0916667 | 1.0646529 | up | HMGA2 |
| 243982_at | 2.093299 | 1.0657783 | up |  |
| 1556499_s_at | 2.093435 | 1.0658722 | up | COL1A1 |
| 1552711_a_at | 2.0987115 | 1.0695038 | up | CYB5D1 |
| 228110_x_at | 2.100204 | 1.0705295 | up | RP11-792A8.4 |
| 225855_at | 2.1008608 | 1.0709805 | up | EPB41L5 |
| 211896_s_at | 2.101788 | 1.0716171 | up | DCN |
| 229685_at | 2.106041 | 1.0745335 | up | LOC100134937 |
| 1556321_a_at | 2.1079519 | 1.0758419 | up | MESDC1 |
| 242904_x_at | 2.10856 | 1.0762582 | up | RP11-489E7.4 |
| 242108_at | 2.1118975 | 1.0785398 | up |  |
| 244411_at | 2.112012 | 1.078618 | up | LOC100507316 |
| 236462_at | 2.1128669 | 1.0792019 | up |  |
| 235271_s_at | 2.1128674 | 1.0792022 | up | ZNF397 |
| 211813_x_at | 2.1152856 | 1.0808525 | up | DCN |
| 243063_at | 2.1167195 | 1.08183 | up |  |
| 228346_at | 2.1170986 | 1.0820885 | up | ZNF844 |
| 235206_at | 2.1192422 | 1.0835485 | up |  |
| 225911_at | 2.1211133 | 1.0848217 | up | NPNT |
| 218880_at | 2.122297 | 1.0856266 | up | FOSL2 |
| 225557_at | 2.1230688 | 1.0861511 | up | CSRNP1 |
| 210281_s_at | 2.125685 | 1.0879278 | up | ZMYM2 |
| 238534_at | 2.1343443 | 1.0937929 | up | LRRFIP1 |
| 204926_at | 2.135412 | 1.0945144 | up | INHBA |
| 201426_s_at | 2.1415448 | 1.0986519 | up | VIM |
| 213679_at | 2.1429653 | 1.0996084 | up | TTC30A |
| 241972_at | 2.1447835 | 1.100832 | up | ZNF674-AS1 |
| 1558111_at | 2.1451054 | 1.1010485 | up | MBNL1 |
| 236170_x_at | 2.1462903 | 1.1018453 | up | RP11-379H18.1 |
| 231417_at | 2.146853 | 1.1022234 | up |  |
| 232839_at | 2.148514 | 1.1033392 | up | STK24-AS1 |
| 1569484_s_at | 2.1485143 | 1.1033394 | up | MDN1 |
| 238893_at | 2.1527483 | 1.1061797 | up | LINC00936 |
| 212143_s_at | 2.1540167 | 1.1070294 | up | IGFBP3 |
| 213916_at | 2.1554146 | 1.1079655 | up | ZNF20///ZNF625-ZNF20 |
| 238624_at | 2.1601195 | 1.1111112 | up | LOC102724517///NLK |
| 232530_at | 2.1622531 | 1.1125355 | up | PLD1 |
| 207624_s_at | 2.1627278 | 1.1128521 | up | RPGR |
| 1559102_at | 2.1630745 | 1.1130834 | up | RP11-73K9.2 |
| 211506_s_at | 2.171182 | 1.1184807 | up | CXCL8 |
| 227410_at | 2.1714096 | 1.1186318 | up | FAM43A |
| 214539_at | 2.1718545 | 1.1189275 | up | SERPINB10 |
| 203184_at | 2.1785486 | 1.1233673 | up | FBN2 |
| 201893_x_at | 2.1800756 | 1.1243782 | up | DCN |
| 205330_at | 2.1801794 | 1.1244469 | up | MN1 |
| 241492_at | 2.180534 | 1.1246815 | up |  |
| 234153_at | 2.1810334 | 1.1250119 | up |  |
| 230450_at | 2.1893184 | 1.1304817 | up | LOC101928545 |
| 229511_at | 2.1901708 | 1.1310434 | up | SMARCE1 |
| 209652_s_at | 2.1926973 | 1.1327066 | up | PGF |
| 227966_s_at | 2.1938686 | 1.1334772 | up | CCDC74A///CCDC74B |
| 236124_at | 2.1938868 | 1.1334891 | up | LOC153546 |
| 207980_s_at | 2.1941335 | 1.1336513 | up | CITED2 |
| 215867_x_at | 2.1969032 | 1.1354713 | up | CA12 |
| 210710_at | 2.1986763 | 1.1366353 | up | AGGF1 |
| 244360_at | 2.1987572 | 1.1366882 | up | FBXL17 |
| 1563933_a_at | 2.1996977 | 1.1373053 | up | PLD5 |
| 235719_at | 2.2010388 | 1.1381845 | up | CYP4V2 |
| 236907_at | 2.2052643 | 1.1409516 | up |  |
| 238646_at | 2.2063456 | 1.1416588 | up |  |
| 241495_at | 2.2091556 | 1.1434951 | up | CCNL1 |
| 1564151_at | 2.2122836 | 1.1455364 | up | LOC102724718 |
| 231403_at | 2.2123518 | 1.1455808 | up | TRIO |
| 220328_at | 2.218578 | 1.1496353 | up | PHC3 |
| 243947_s_at | 2.2288928 | 1.1563272 | up |  |
| 1554287_at | 2.2293327 | 1.1566119 | up | TRIM4 |
| 244498_x_at | 2.2357 | 1.1607265 | up | UBE2Q2L |
| 225582_at | 2.2372959 | 1.161756 | up | ITPRIP |
| 233019_at | 2.2386107 | 1.1626036 | up | CNOT7 |
| 1555978_s_at | 2.2493615 | 1.1695156 | up | MYL12A |
| 235094_at | 2.251419 | 1.1708345 | up |  |
| 228049_x_at | 2.257462 | 1.1747017 | up | SNHG19 |
| 1555920_at | 2.2593968 | 1.1759377 | up | CBX3 |
| 235490_at | 2.2595074 | 1.1760082 | up | TMEM107 |
| 1558052_at | 2.2642357 | 1.1790242 | up | TMED4 |
| 241036_at | 2.2703629 | 1.1829228 | up |  |
| 243531_at | 2.2756126 | 1.186255 | up | ORAOV1 |
| 236245_at | 2.2774637 | 1.187428 | up | ODF3L1 |
| 238657_at | 2.2780297 | 1.1877866 | up | UBXN10 |
| 236947_at | 2.278859 | 1.1883116 | up |  |
| 211032_at | 2.279645 | 1.1888092 | up | COBLL1 |
| 1559532_at | 2.2880256 | 1.1941032 | up | ARIH2OS |
| 207526_s_at | 2.2986739 | 1.2008018 | up | IL1RL1 |
| 1557567_a_at | 2.3013833 | 1.2025013 | up | LOC100507634 |
| 217208_s_at | 2.3017302 | 1.2027187 | up | DLG1 |
| 239123_at | 2.3022475 | 1.203043 | up | TSC22D1 |
| 240259_at | 2.3081334 | 1.2067266 | up | FLRT2///LOC100506718///LOC102724348 |
| 213718_at | 2.3099694 | 1.2078738 | up | RBM14-RBM4///RBM4 |
| 1559970_at | 2.3248694 | 1.2171497 | up | LOC100506731 |
| 236662_at | 2.3298633 | 1.2202454 | up |  |
| 1559009_at | 2.3382497 | 1.225429 | up | LOC101928504 |
| 236948_x_at | 2.3394449 | 1.2261662 | up | SRSF11 |
| 220468_at | 2.3425918 | 1.2281055 | up | ARL14 |
| 1553299_at | 2.3431222 | 1.2284322 | up | DUSP5P1 |
| 230542_at | 2.3598812 | 1.2387142 | up | ZNF597 |
| 229756_at | 2.3645344 | 1.2415562 | up | LOC100506299 |
| 243977_at | 2.3649683 | 1.2418208 | up | LOC541472 |
| 228181_at | 2.367891 | 1.2436028 | up | SLC30A1 |
| 230636_s_at | 2.3692126 | 1.2444077 | up | KLF9 |
| 228097_at | 2.3717644 | 1.2459607 | up | MYLIP |
| 240165_at | 2.3719456 | 1.2460709 | up |  |
| 215078_at | 2.3807437 | 1.2514124 | up | LOC100129518///SOD2 |
| 236115_at | 2.3808494 | 1.2514763 | up | HTR7P1 |
| 1558053_s_at | 2.3818347 | 1.2520733 | up | TMED4 |
| 226458_at | 2.3881314 | 1.2558823 | up | RP1-39G22.7 |
| 241710_at | 2.3884962 | 1.2561026 | up | LOC728819 |
| 209167_at | 2.388976 | 1.2563925 | up | GPM6B |
| 235428_at | 2.3905852 | 1.2573638 | up | LOC100507316 |
| 1554906_a_at | 2.4150128 | 1.2720308 | up | MPHOSPH6 |
| 231285_at | 2.417749 | 1.2736645 | up |  |
| 239081_at | 2.4256604 | 1.2783775 | up |  |
| 236591_at | 2.4296024 | 1.2807202 | up | MIR4458///MIR4458HG |
| 1557285_at | 2.4315333 | 1.2818663 | up | AREG |
| 237290_at | 2.4343727 | 1.28355 | up |  |
| 237291_at | 2.4368105 | 1.2849941 | up | PRORSD1P |
| 1555441_at | 2.4392684 | 1.2864485 | up | UBA6 |
| 238012_at | 2.4416463 | 1.2878542 | up | DPP7 |
| 209335_at | 2.4420612 | 1.2880993 | up | DCN |
| 241823_at | 2.448156 | 1.2916956 | up |  |
| 202912_at | 2.4516494 | 1.2937527 | up | ADM |
| 223185_s_at | 2.4561865 | 1.2964201 | up | BHLHE41 |
| 240355_at | 2.4588938 | 1.2980094 | up |  |
| 1552634_a_at | 2.4638457 | 1.3009119 | up | ZNF101 |
| 233159_at | 2.464499 | 1.3012943 | up | STARD13-AS |
| 238623_at | 2.4681475 | 1.3034286 | up | RP3-428L16.2 |
| 242045_at | 2.4747577 | 1.3072872 | up |  |
| 237028_at | 2.4770334 | 1.3086133 | up | ENO1-AS1 |
| 218820_at | 2.4793735 | 1.3099756 | up | C14orf132 |
| 238766_at | 2.502083 | 1.3231297 | up |  |
| 1557117_at | 2.502811 | 1.3235493 | up | INTS6-AS1 |
| 1554036_at | 2.504893 | 1.324749 | up | ZBTB24 |
| 240038_at | 2.5120513 | 1.328866 | up |  |
| 239876_at | 2.5168767 | 1.3316345 | up |  |
| 1554037_a_at | 2.52969 | 1.3389606 | up | ZBTB24 |
| 230991_at | 2.533482 | 1.3411217 | up | LOC102724156 |
| 244026_at | 2.5338275 | 1.3413184 | up |  |
| 242853_at | 2.5504956 | 1.3507776 | up |  |
| 1555325_s_at | 2.5550365 | 1.353344 | up | ZNF26 |
| 1555912_at | 2.5655425 | 1.3592639 | up | ST7-AS1 |
| 236937_at | 2.5856385 | 1.3705206 | up | VPS8 |
| 226534_at | 2.5907884 | 1.3733912 | up | KITLG |
| 235879_at | 2.606638 | 1.3821902 | up |  |
| 241786_at | 2.615816 | 1.3872612 | up |  |
| 243871_at | 2.6159048 | 1.38731 | up | LOC100130476 |
| 228820_at | 2.6327024 | 1.3965445 | up | XPNPEP3 |
| 207113_s_at | 2.6418865 | 1.4015684 | up | TNF |
| 1559535_s_at | 2.6434052 | 1.4023976 | up | BC040901///CTC-241N9.1///LOC100996419 |
| 239343_at | 2.6570456 | 1.409823 | up | LOC728705///TMEM263 |
| 241773_at | 2.6627944 | 1.412941 | up |  |
| 203963_at | 2.6706493 | 1.4171906 | up | CA12 |
| 230738_at | 2.6732872 | 1.4186149 | up | RP11-196G18.23 |
| 231947_at | 2.6975634 | 1.4316568 | up | MYCT1 |
| 1556676_a_at | 2.7023108 | 1.4341936 | up | RP11-425D10.10 |
| 220148_at | 2.7137702 | 1.4402986 | up | ALDH8A1 |
| 1556195_a_at | 2.7260458 | 1.4468098 | up | RP4-798A10.7 |
| 235219_at | 2.7336066 | 1.4508057 | up | C5orf55 |
| 237051_at | 2.7391472 | 1.4537268 | up |  |
| 1556361_s_at | 2.7457495 | 1.4572 | up | ANKRD13C |
| 209795_at | 2.7460895 | 1.4573786 | up | CD69 |
| 211466_at | 2.748766 | 1.4587841 | up | NFIB |
| 210941_at | 2.757489 | 1.4633551 | up | PCDH7 |
| 242865_at | 2.7956908 | 1.4832048 | up |  |
| 206157_at | 2.8017812 | 1.4863443 | up | PTX3 |
| 204748_at | 2.8046184 | 1.4878044 | up | PTGS2 |
| 221530_s_at | 2.8558729 | 1.5139318 | up | BHLHE41 |
| 206522_at | 2.8559573 | 1.5139744 | up | MGAM |
| 1554997_a_at | 2.8560474 | 1.51402 | up | PTGS2 |
| 236079_at | 2.8581808 | 1.5150971 | up | LOC202025 |
| 215446_s_at | 2.878654 | 1.5253944 | up | LOX |
| 206969_at | 2.9242692 | 1.5480762 | up | KRT34///LOC100653049 |
| 243296_at | 2.9263973 | 1.5491257 | up | NAMPT |
| 236174_at | 2.9386623 | 1.5551596 | up | RP11-79P5.9 |
| 213640_s_at | 2.9543786 | 1.5628548 | up | LOX |
| 206685_at | 2.965753 | 1.5683985 | up | HCG4 |
| 1557488_at | 2.9886334 | 1.5794859 | up | CBX3P2 |
| 231952_at | 2.9991748 | 1.5845656 | up |  |
| 1558345_a_at | 3.0008466 | 1.5853696 | up | LOC439911 |
| 232964_at | 3.0269766 | 1.5978775 | up | SPDYE1///SPDYE2///SPDYE2B///SPDYE5///SPDYE6 |
| 238633_at | 3.0399191 | 1.604033 | up | EPC1 |
| 1557257_at | 3.0537145 | 1.6105652 | up | BCL10 |
| 209101_at | 3.086049 | 1.625761 | up | CTGF |
| 238389_s_at | 3.0892525 | 1.6272578 | up |  |
| 210355_at | 3.108986 | 1.6364441 | up | PTHLH |
| 237309_at | 3.129014 | 1.6457081 | up |  |
| 1569411_at | 3.1324003 | 1.6472685 | up | TMEM67 |
| 211756_at | 3.1484768 | 1.654654 | up | PTHLH |
| 204818_at | 3.1562881 | 1.6582289 | up | HSD17B2 |
| 204298_s_at | 3.2612462 | 1.7054234 | up | LOX |
| 230598_at | 3.2644124 | 1.7068233 | up | RP11-554J4.1 |
| 209737_at | 3.285825 | 1.7162557 | up | MAGI2 |
| 1556216_s_at | 3.3148549 | 1.7289457 | up | BC042372///RP11-45P15.4 |
| 1557118_a_at | 3.3306892 | 1.7358208 | up | INTS6-AS1 |
| 227099_s_at | 3.3430316 | 1.741157 | up | C11orf96 |
| 1553099_at | 3.361233 | 1.7489905 | up | TIGD1 |
| 230307_at | 3.3616385 | 1.7491646 | up | SLC25A21-AS1 |
| 236213_at | 3.3870838 | 1.7600436 | up | AC079305.10 |
| 1564653_s_at | 3.4620645 | 1.7916327 | up | LEKR1 |
| 242255_at | 3.498544 | 1.8067546 | up | WDR37 |
| 222310_at | 3.5023 | 1.8083026 | up | SCAF4 |
| 1554309_at | 3.5444517 | 1.8255625 | up | EIF4G3 |
| 1557164_a_at | 3.5749357 | 1.8379173 | up |  |
| 231709_x_at | 3.638508 | 1.863347 | up |  |
| 223698_at | 3.7404172 | 1.9031992 | up | SLC25A36 |
| 203603_s_at | 3.7481544 | 1.9061804 | up | ZEB2 |
| 1559975_at | 3.7725124 | 1.9155257 | up | BTG1 |
| 209170_s_at | 3.927299 | 1.9735374 | up | GPM6B |
| 218723_s_at | 4.2093487 | 2.073597 | up | RGCC |
| 230748_at | 4.2160068 | 2.0758772 | up | SLC16A6 |
| 206300_s_at | 4.2445436 | 2.0856094 | up | PTHLH |
| 205681_at | 4.7640376 | 2.2521849 | up | BCL2A1 |
| 240013_at | 5.1525593 | 2.3652892 | up |  |
| 1556773_at | 5.555655 | 2.473957 | up |  |
| 224797_at | 5.9244976 | 2.5666928 | up | ARRDC3 |
| 207850_at | 6.5672097 | 2.7152805 | up | CXCL3 |

**Supplementary Figure Legends**

**Figure S1.** Box plot showing the expression of the GLUT family members correlated with the survival rate of the patients in the Petel HNSCC cohort (E-MTAB-1328, n=89) in the SurvExpress database (HR=3.37, *P*=0.043).

**Figure S2.** Forest plot of GLUT family members and their corresponding hazard ratios, probes and Cox-P values.

**Figure S3.** (A) Cell proliferation rate *in vitro* and (B) tumorigenicity ability *in vivo* of GLUT4-overexpressing FaDu cells.

**Figure S4.** Glucose uptake and lactate production in a panel of HNSCC cell lines.

**Figure S5.** The migration abilities of with or without GLUT4 knockdown combined DDX58 or OASL knockdown in HSC-2 cells.

**Figure S6.** Correlation plot of GLUT4 expression with the (A) OASL or (B) DDX58 RNA level in a clinical cohort (Pearson r= -0.7146, *P*<0.001 and Pearson r= -0.6246, *P*<0.001, respectively).

**
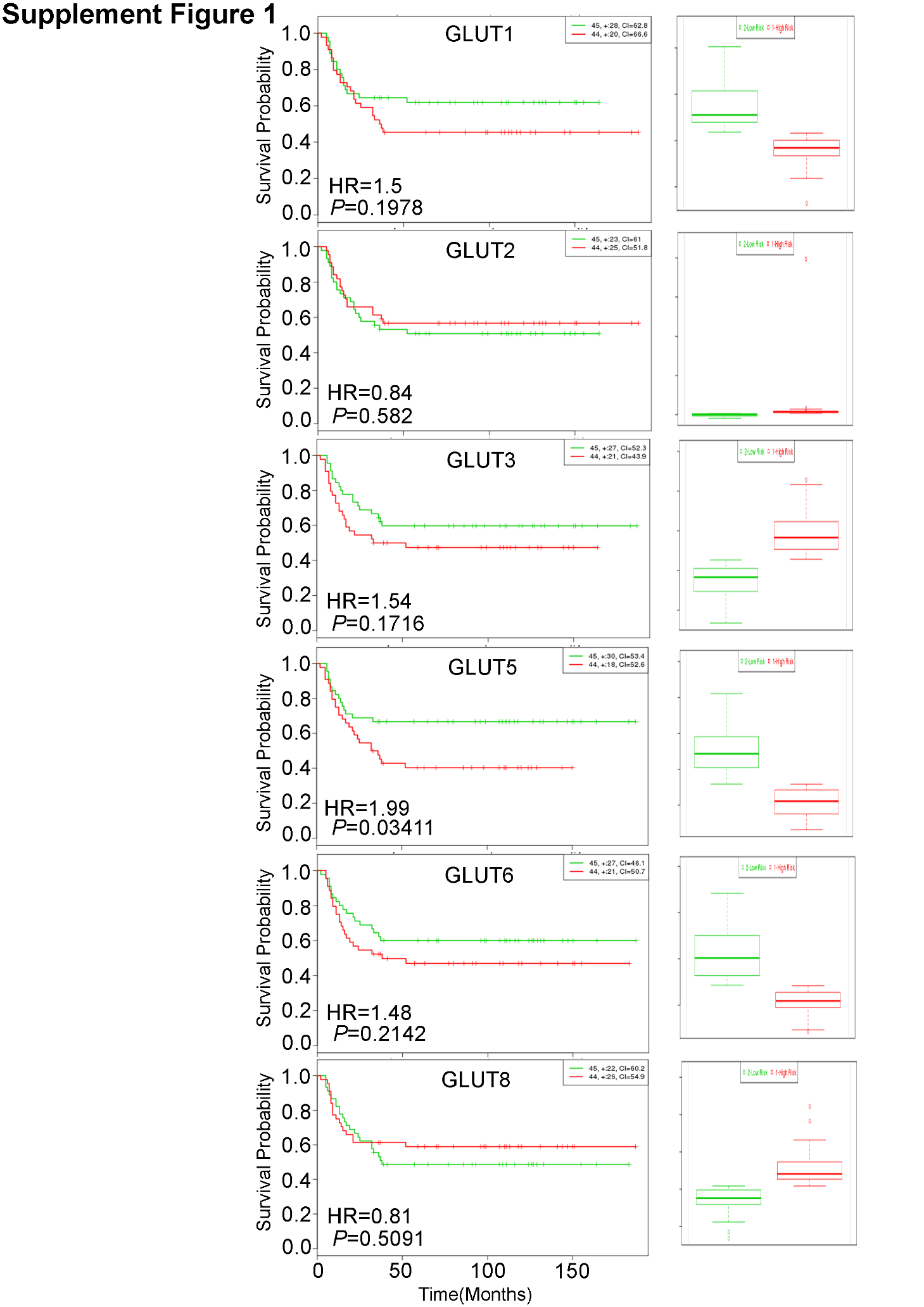
**


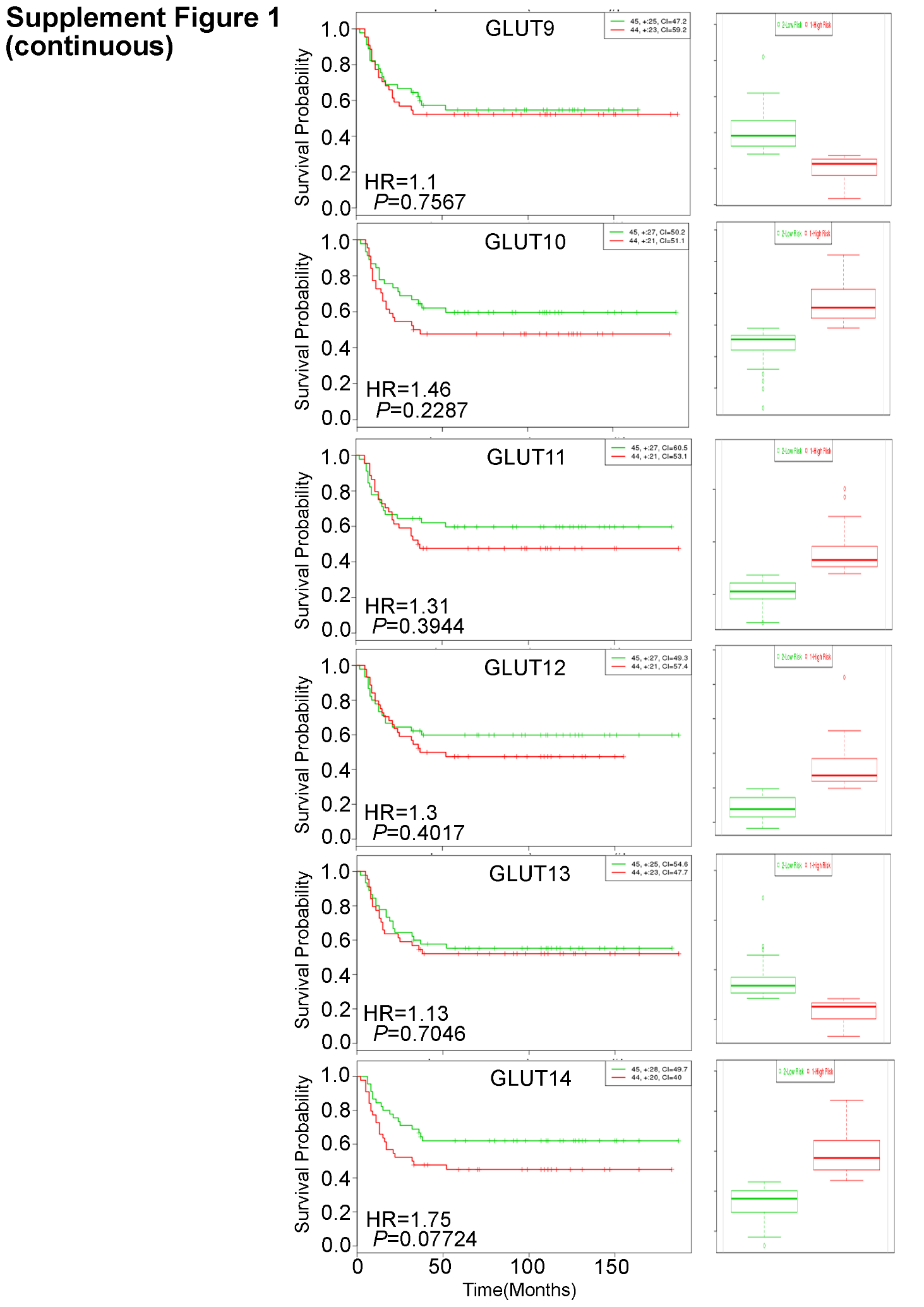


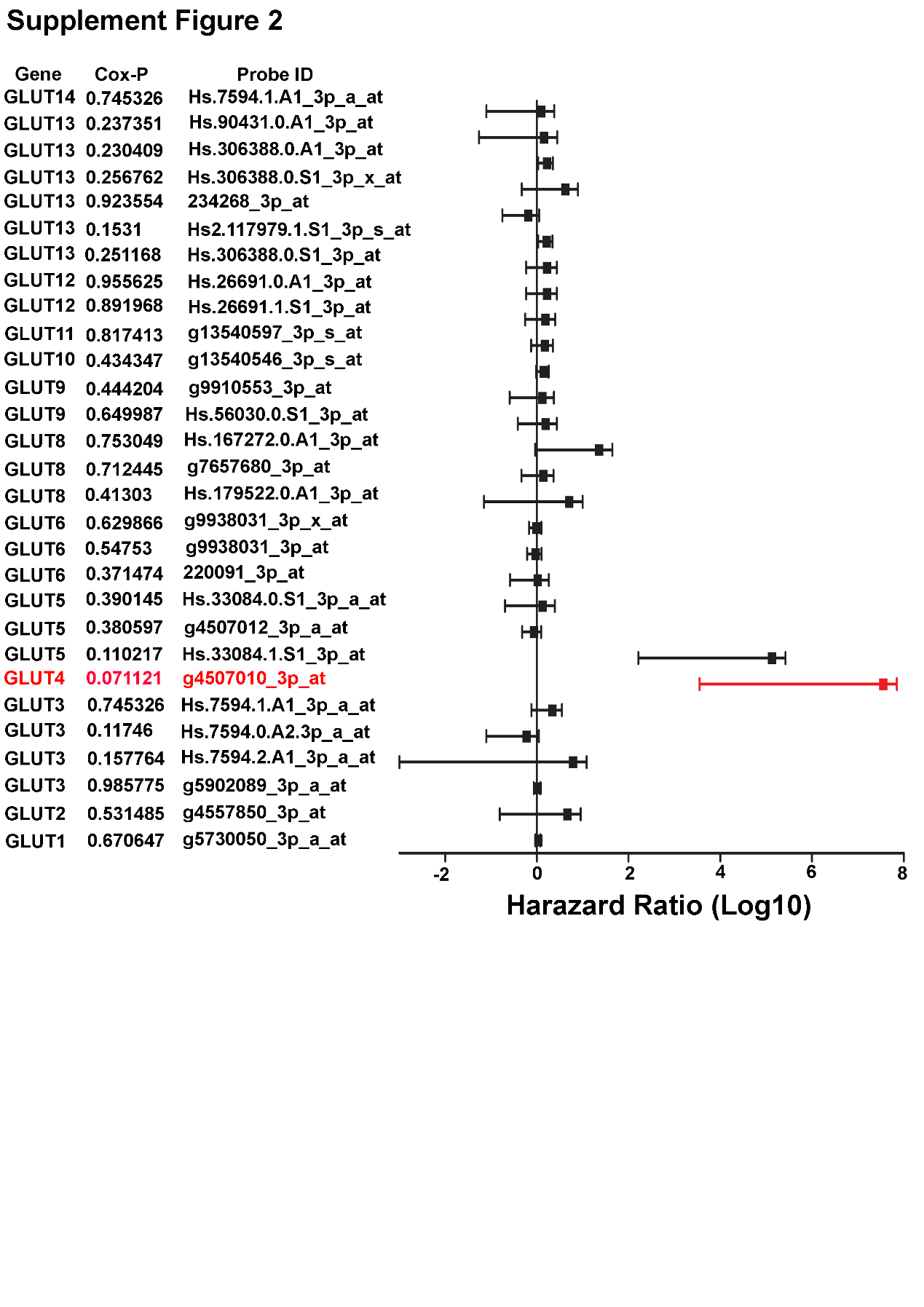

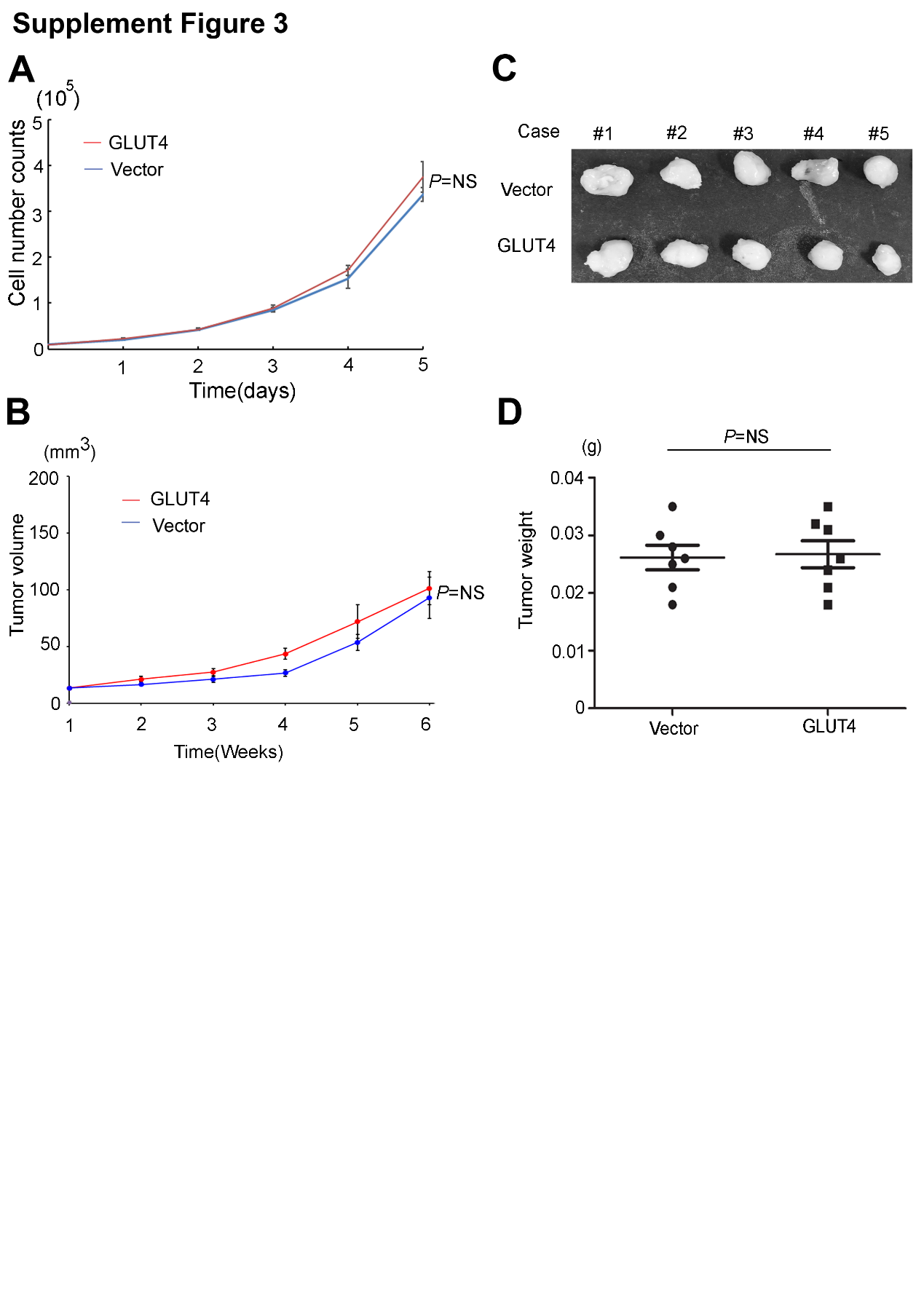

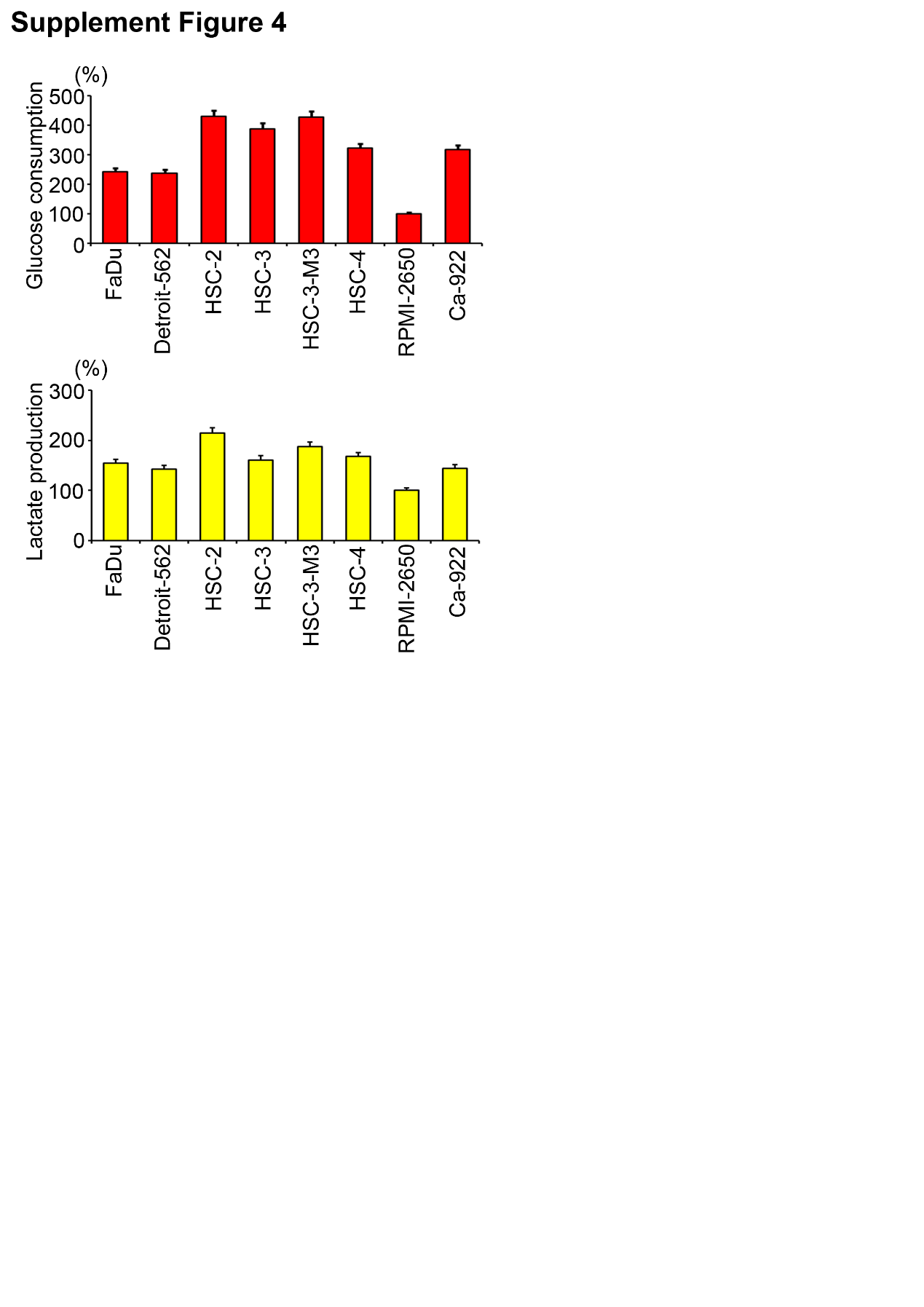

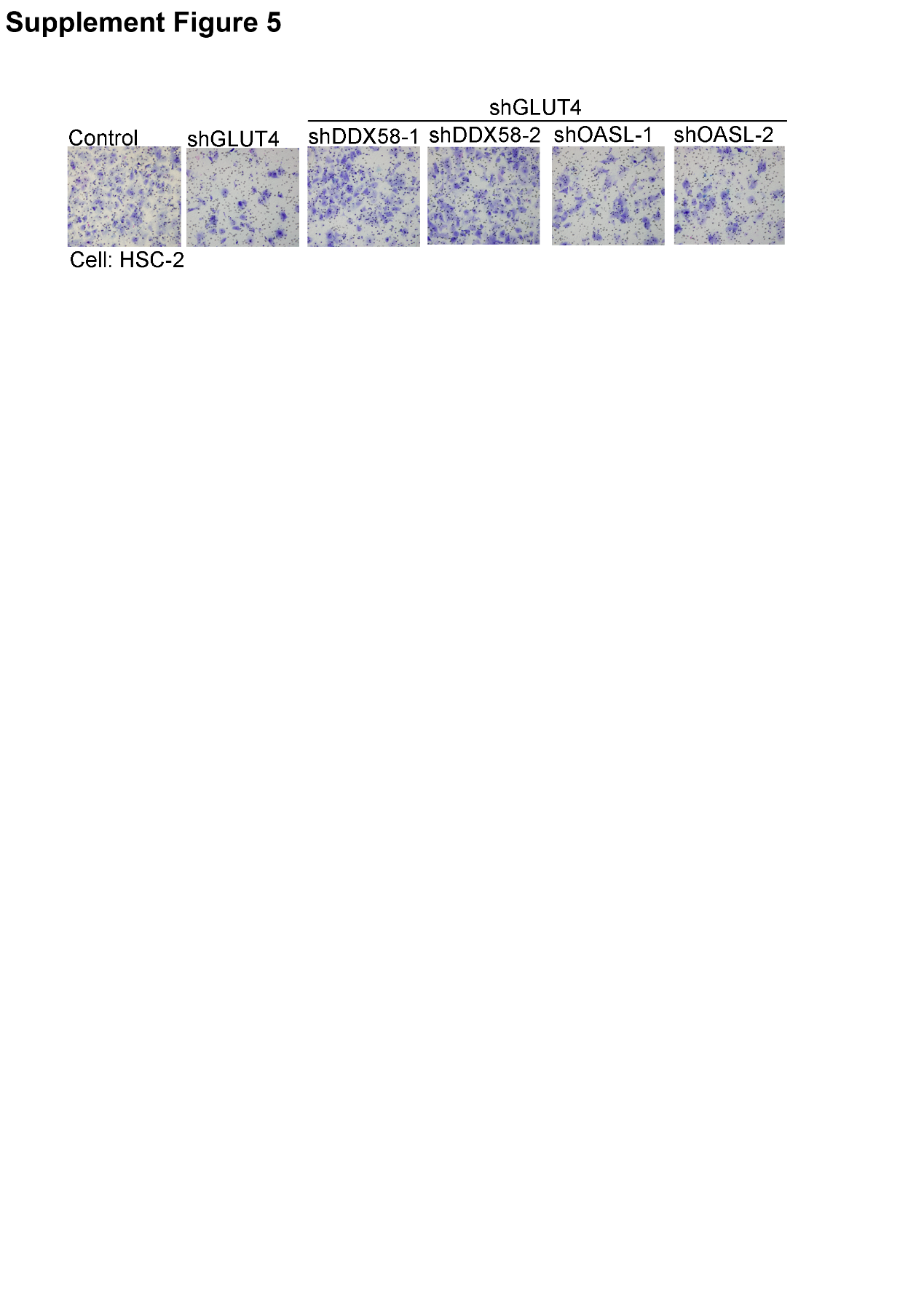

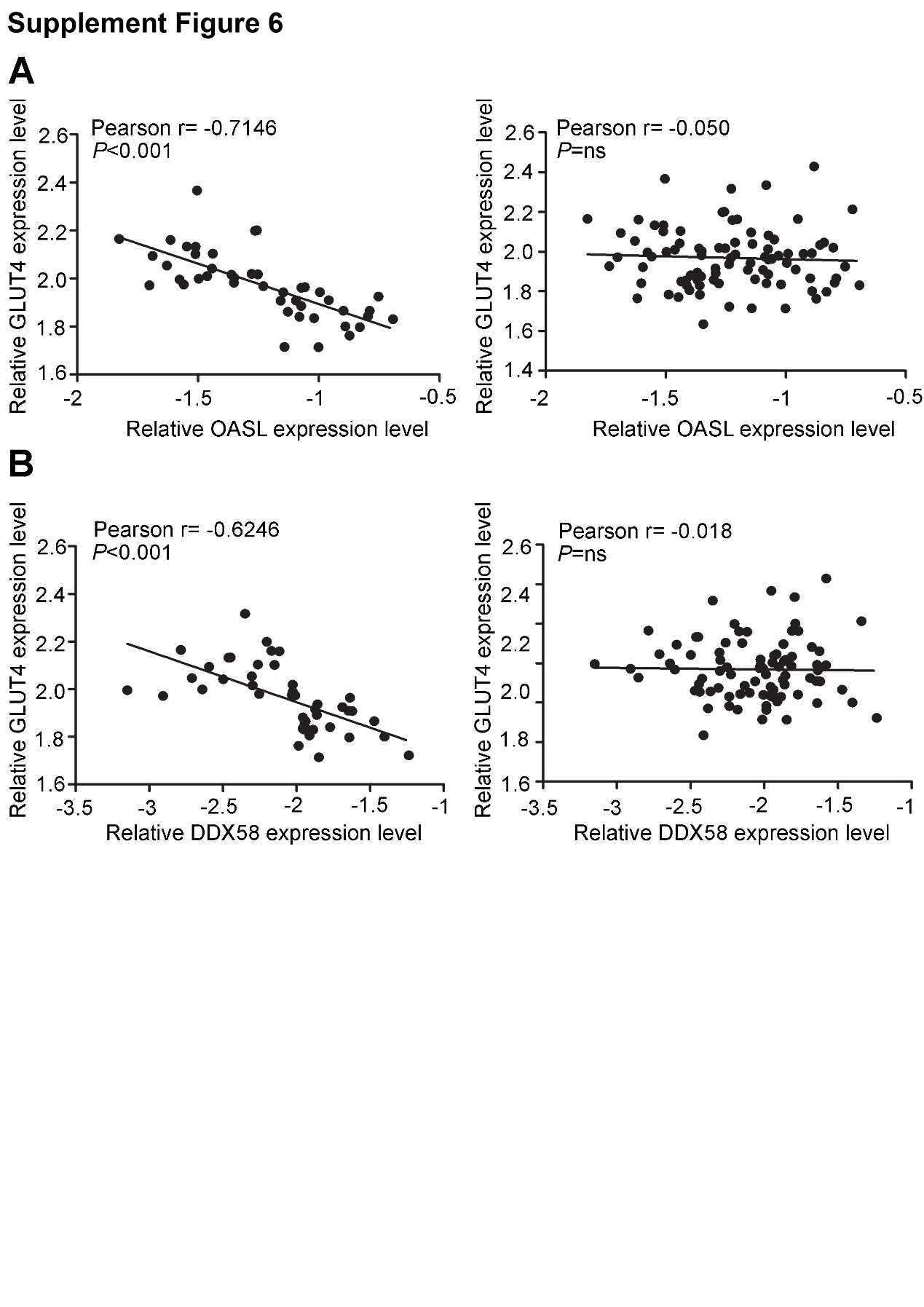

Supplement: Additional file 1: Table S1. — Demographic features of HNCC patient cohort. Table S2. GLUT overexpression activated transcription factors and their downstream targets ranked by Z-Score. Table S3. GLUT overexpression inhibited transcription factors and their downstream targets ranked by Z-Score. Table S4. List of TRIM24 downstream genes and their fold changes. Table S5. List of primers and knockdown clones’ information. Table S6. List of candidate probes >2.0-fold change cutoff by GLUT4 vs. control in FaDu cells. Figure S1. Box plot showing the expression of the GLUT family members correlated with the survival rate of the patients in the Petel HNSCC cohort (E-MTAB-1328, n = 89) in the SurvExpress database (HR = 3.37, P = 0.043). Figure S2. Forest plot of GLUT family members and their corresponding hazard ratios, probes and Cox-P values. Figure S3. GLUT4 overexpression model in vitro and in vivo. (A) Cell proliferation rate and (B) tumorigenicity ability in animal model GLUT4-overexpressing FaDu cells. Figure S4. Glucose uptake and lactate production in a panel of HNSCC cell lines. Figure S5. The migration abilities of with or without GLUT4 knockdown combined DDX58 or OASL knockdown in HSC-2 cells. Figure S6. Correlation plot of GLUT4 expression with the (A) OASL or (B) DDX58 RNA level in a clinical cohort (Pearson r = −0.7146, P < 0.001 and Pearson r = −0.6246, P < 0.001, respectively). (DOCX 2697 kb) [file 13045_2016_372_MOESM1_ESM.docx]
